# Using real-world data to predict findings of an ongoing phase IV trial: glycemic control of semaglutide versus standard of care

**DOI:** 10.1136/bmjdrc-2025-005180

**Published:** 2025-10-29

**Authors:** Sushama Kattinakere Sreedhara, Sebastian Schneeweiss, Elvira D’Andrea, Janick G Weberpals, Elyse C DiCesare, Elisabetta Patorno, Theodore Tsacogianis, Marie Bradley, John Concato, Shirley V Wang

**Affiliations:** 1 Division of Pharmacoepidemiology and Pharmacoeconomics, Department of Medicine, Brigham and Women’s Hospital, Boston, Massachusetts, USA; 2 Office of Medical Policy, Center for Drug Evaluation and Research, US Food and Drug Administration, Silver Spring, Maryland, USA; 3 Department of Medicine, Yale School of Medicine, New Haven, Connecticut, USA

**Keywords:** Cohort Studies, Database, Pharmacoepidemiology, Glycated Hemoglobin A

## Abstract

**Objective:**

Using national claims databases, we sought to emulate the design of the ongoing SEPRA trial and predict its findings, comparing the effects of once weekly semaglutide to SoC medications on glycemic control (A1C <7%) in type-2 diabetes mellitus (T2D).

**Research design and methods:**

Using Optum Clinformatics (July 2017 – May 2022), we identified a 1:1 propensity score-matched (PSM) cohort of adults with T2D on metformin monotherapy, who had recorded A1C and initiated either injectable semaglutide or SoC medications (dipeptidyl peptidase-4 inhibitors, sodium-glucose cotransporter-2 inhibitors, SUs, or glucagon-like peptide-1 agonists) and met eligibility criteria adapted from the SEPRA trial. The primary outcome was the proportion of patients achieving A1C <7%. The study protocol was preregistered (NCT05577728, ClinicalTrials.gov) before any etiologic analyses. Risk ratios and corresponding 95% CIs were estimated.

**Results:**

We identified 1,144 PSM pairs of injectable semaglutide and SoC initiators with balance in pre-exposure covariates. Semaglutide initiators were 30% (risk ratio (95% CI), 1.30 (1.16 to 1.45)) more likely to achieve glycemic control (A1C <7%) than those initiating SoC. Additionally, semaglutide initiators had a 1.3% reduction in A1C, compared with a 1.1% reduction in the SoC group. These results were consistent with interim results of the SEPRA trial, which were released after the protocol for our database study was preregistered.

**Conclusion:**

This claims database study, designed to predict the results of the SEPRA trial, found results consistent with interim trial results. Our findings support the notion that well-designed non-randomized studies using fit-for-purpose data can effectively complement pragmatic randomized controlled trials.

**WHAT IS ALREADY KNOWN ON THIS TOPIC**

- The SEmaglutide PRAgmatic (SEPRA) trial was launched to evaluate the efficacy of once-weekly injectable semaglutide compared with standard of care in improving glycemic control among patients with type two diabetes mellitus.
- While randomized trials provide robust evidence, there is increasing interest in using real-world evidence studies to complement evidence from trials.

**WHAT THIS STUDY ADDS**

- We emulated the design of the ongoing SEPRA trial and predicted the results in a study conducted using real-world data.
- We found that patients initiating semaglutide were 30% more likely to achieve glycemic control (glycated hemoglobin <7%) compared with those receiving standard of care.
- The study’s findings closely align with interim results from the ongoing SEPRA trial.

**HOW THIS STUDY MIGHT AFFECT RESEARCH, PRACTICE, OR POLICY**

- This study illustrates how real-world evidence studies can complement clinical trials and provide timely evidence to inform decision-making.
- The findings support the broader integration of real-world evidence into the development of treatment guidelines as well as regulatory and reimbursement decisions.

## Introduction

 Although traditional randomized controlled trials (RCTs) are considered a key tool for testing the efficacy of medical products,^1 2^ it is not feasible to conduct trials for every question in every population. Consequently, there is growing interest among medical and regulatory communities in the USA in using real-world data (RWD) and real-world evidence (RWE) to address drug safety and effectiveness.^3^,^7^ Under the US Food and Drug Administration’s RWE Program, established by the 21st Century Cures Act,^8^ data collected during routine clinical care—such as longitudinal electronic health records and claims databases—are considered RWD.^9^ While questions persist regarding the reliability of RWD analysis for regulatory decision-making on drug safety and effectiveness, investigators have successfully emulated RCT findings using non-interventional studies in select indications.^10^,^13^ Concordance in results with completed trials can increase confidence in the ability of database studies to come to causal conclusions, particularly when such studies emulate trial protocols prospectively and produce findings similar to those of ongoing randomized trials whose results are not yet public. Although observational studies may not establish causality on their own, principled designs aimed at minimizing bias and confounding can strengthen the validity of causal interpretations, as demonstrated in recent efforts to emulate multiple RCTs using RWD.^13^

The SEmaglutide PRAgmatic (SEPRA) clinical trial is an ongoing, randomized, open-label, pragmatic clinical trial designed to compare the effectiveness of treatment intensification of current antidiabetic therapy with either once-weekly subcutaneous semaglutide or any other non-insulin diabetes treatment in adults with type 2 diabetes mellitus (T2DM) in clinical practice in the USA.^14 15^ This pragmatic trial, as assessed by the PRagmatic Explanatory Continuum Indicator Summary (PRECIS) criteria,^15 16^ had minimal eligibility criteria and allowed treating physicians to make decisions about treatment changes, discontinuation, and augmentation after randomization, as they would in routine clinical care.

The American Diabetes Association guidelines at the time of the SEPRA trial recommended metformin as first-line therapy and second-line treatments if glycated hemoglobin (A1C) levels exceed 7% with agents such as dipeptidyl peptidase-4 inhibitors (DPP-4is), sodium-glucose cotransporter-2 inhibitors (SGLT-2is), or sulfonylurea (SUs), or glucagon-like peptide-1 receptor agonists (GLP-1RAs), considering other comorbidities, atherosclerotic cardiovascular disease, chronic kidney disease, patient preferences, and other social determinants of health.^15 17^

We conducted a non-interventional study using RWD to predict SEPRA trial results. This work is part of a broader series of studies using RWD designed to predict the results of ongoing trials before the results are available.^11 18^

## Research design and methods

### Data source

We implemented this study using Optum’s deidentified Clinformatics Data Mart Database (July 2017 – May 2022) which includes commercial health plan data and Medicare Advantage members. This geographically diverse database spans all 50 states and provides comprehensive demographic information, enrollment status, inpatient and outpatient diagnoses, as well as procedure codes in the form of International Classification of Diseases, Current Procedural Terminology, and Healthcare Common Procedure Coding System codes. Information was also available on pharmacy dispensing records, including information on medication start and refill, strength, quantity, and days’ supply, for insured patients. Outpatient laboratory test results are available for a subset of beneficiaries whose labs are processed by a nationally operating laboratory test provider.

### Study population and treatment

Emulating the SEPRA trial design,^14 15^ we established a cohort of adult T2DM patients who are on metformin monotherapy and who had a record of A1C either starting injectable semaglutide or a standard of care (SoC) antidiabetic medication. SoC included DPP-4is, SGLT-2is, or SUs, or GLP-1RAs other than injectable semaglutide, including exenatide.

The cohort entry date (CED) was defined as the dispensation date of injectable semaglutide (exposure) or SoC medications (comparator) between December 6, 2017 (following the approval of injectable semaglutide in the USA),^19 20^ and May 31, 2021. All eligible patients were required to have 180 days of continuous medical and pharmacy enrollment, during which there was no dispensation of either exposure or comparator medications, indicating new initiators.

The initial eligibility criteria for the SEPRA trial required patients to be on metformin monotherapy, excluding those using other antidiabetic medications. These eligibility criteria underwent multiple amendments, importantly allowing patients to be on one or two oral antidiabetic medications before being randomized. Given that the SEPRA trial protocol amendments were not public at the time that we preregistered our study protocol, the emulation’s eligibility criteria were designed to mimic the initial SEPRA trial eligibility criteria: being 18 years or older, having a diagnosis of T2DM and A1C value, being treated with metformin monotherapy, and started intensification of treatment to achieve glycemic control. Similar to the SEPRA trial, we excluded patients who were pregnant or had evidence of contraindications for semaglutide, including a record for advanced CKD, end-stage renal disease, dialysis, or renal transplant. More details are provided in online supplemental figure S1 and table S1, and operational details of the algorithms at https://osf.io/7smp9.

### Pre-exposure patient characteristics

We assessed >50 patient characteristics in the baseline period of 180 days before the CED. The assessed covariates included demographic variables, calendar time, baseline A1C value, frailty score,^21^ combined comorbidity score,^22 23^ diabetes-specific complications, other comorbidities, use of medications, healthcare utilization indicators as a proxy for overall disease state, care intensity, and surveillance. A full list of covariates is provided in online supplemental table S2.

### Outcomes

We emulated the trial’s primary outcome: the proportion of patients achieving A1C <7% at the end of year 1. We also assessed two secondary outcomes from the trial: the change in A1C (percentage points) from baseline to year one and the number of severe hypoglycemic episodes leading to inpatient admission or emergency room encounters, consistent with the trial definition of severe hypoglycemia. We assessed hypoglycemia based on diagnosis codes^24 25^ indicating hypoglycemia (Positive predicitive value (PPV) between 70–80%), either as the primary diagnosis in inpatient care settings or in any position in emergency department (ED) settings. The algorithm used is provided at https://osf.io/7smp9.

### Follow-up

To mimic the SEPRA design, which allowed treatment intensification, switching, and discontinuation at the treating physician’s discretion, we conducted an as-started (AS) analysis as the observational analog to the intention-to-treat of the trial analysis as our primary causal contrast.^15^ For the A1C outcomes (ie, A1C <7% and change in A1C), the A1C value was evaluated in the 1-year outcome assessment window, that is, 275–455 days after CED, using the closest value to 365 days (online supplemental figure S2). The follow-up started from the beginning of the outcome assessment window and continued until the occurrence of the outcome, disenrollment from the health plan, death, nursing home admission, or the end of the study period. For the number of hypoglycemic episode outcomes, follow-up started 1 day after CED and continued until the occurrence of the outcome, disenrollment from the health plan, death, nursing home admission, or the end of the study period (May 31, 2022), whichever came first (online supplemental figure S3).

To mimic the high level of adherence usually seen in clinical trials, we secondarily conducted as-treated (AT) analyses as an exploratory secondary analysis. As the patients had an average follow-up time while on the treatment of 150 days in our AT cohort, the outcome assessment window was defined at around 5 months (91–212 days). The follow-up started from the beginning of the outcome assessment window and continued until the discontinuation of the drugs, or switch/augmentation of therapy, the occurrence of the outcome, disenrollment from the health plan, death, nursing home admission, or the end of the study period. We defined discontinuation as a gap of more than 60 days following the last day of supply of the exposure/comparator or metformin therapy. Switch/augmentation was defined as adding other antidiabetic agents beyond the one initiated on the CED (see online supplemental figures S2 and S4).

### Statistical analysis

We used 1:1 nearest-neighbor propensity score (PS) matching without replacement and a caliper of 0.01 of the PS distribution to balance >50 patient characteristics across treatment groups. The PS was estimated by fitting a logistic regression model without further variable selection (online supplemental table S2). https://osf.io/gjmuv provides the R code used to create the PS matching cohort. We assessed the postmatching balance via: (1) the c-statistic, expecting 0.5 for perfect balance in measured patient characteristics, and (2) the standardized difference between the characteristics, ≤0.1 indicating good balance.^26^

After matching, our feasibility counts substantially exceeded the trial’s intended recruitment of 1,387 patients, which was estimated to provide 90% power to confirm the superiority.^14^ Therefore, we inferred that our study would have at least equal power to the SEPRA trial. For the outcomes of A1C <7% and change in A1C, we imputed the missing A1C value for those patients who entered the 1-year outcome assessment window but did not have A1C measurement using multiple imputation by chained equations (MICE) in combination with a random forest approach to imputing missing data (online supplemental methods 1 for details).^27 28^

A detailed protocol of our study was registered on clinicaltrials.gov on October 10, 2022, before conducting inferential analyses. Because SEPRA’s registration did not specify how the proportions of patients with A1C <7% would be compared between the treatment arms, we chose a relative risk estimate.^29 30^ The change in A1C from baseline to follow-up was calculated as the difference between follow-up and baseline A1C values in percent points. All analyses were performed using Aetion Evidence Platform V.5.5 (Aetion), which has previously been validated by accurately repeating a range of previously published studies,^31 32^ STATA V.17 (StataCorp), and R V.4.4.1.

### Subgroup and sensitivity analyses

We conducted several prespecified subgroup analyses, assessing A1C outcomes using each of the four drug classes included in the SoC as separate comparator groups.

To evaluate the robustness of our missing data imputation results, we conducted multiple sensitivity analyses for both our AS and AT causal contrasts. We conducted a complete case analysis where we assessed the A1C outcome only among patients with A1C values in the outcome assessment window. In addition, we used different imputation methods, including MICE^33 34^ and fixed imputation of values at different points of the observed distribution of A1C in follow-up as a tipping point analysis, as well as a delta-adjusted missing not at random sensitivity analysis to test the impact of lower A1C values^35^ (online supplemental methods 2).

### Evaluation of concordance of results between RWD study and RCT

To assess whether trial emulation would lead to the same regulatory conclusions as the interim results of the ongoing RCT, we computed three predefined binary metrics^1^: statistical significance agreement, where estimates and CIs are on the same side of the null^2^; estimate agreement, where the trial emulation estimates fall within the 95% CI of the interim trial results and^3^ standardized difference agreement, which compares the treatment effect estimates between the trials and their emulations.^13 36^ We were able to calculate concordance for our primary outcome (A1C <7%) but could not do so for the change in A1C percentage points due to the lack of SD reporting in the interim results.

## Results

**Figure 1:**
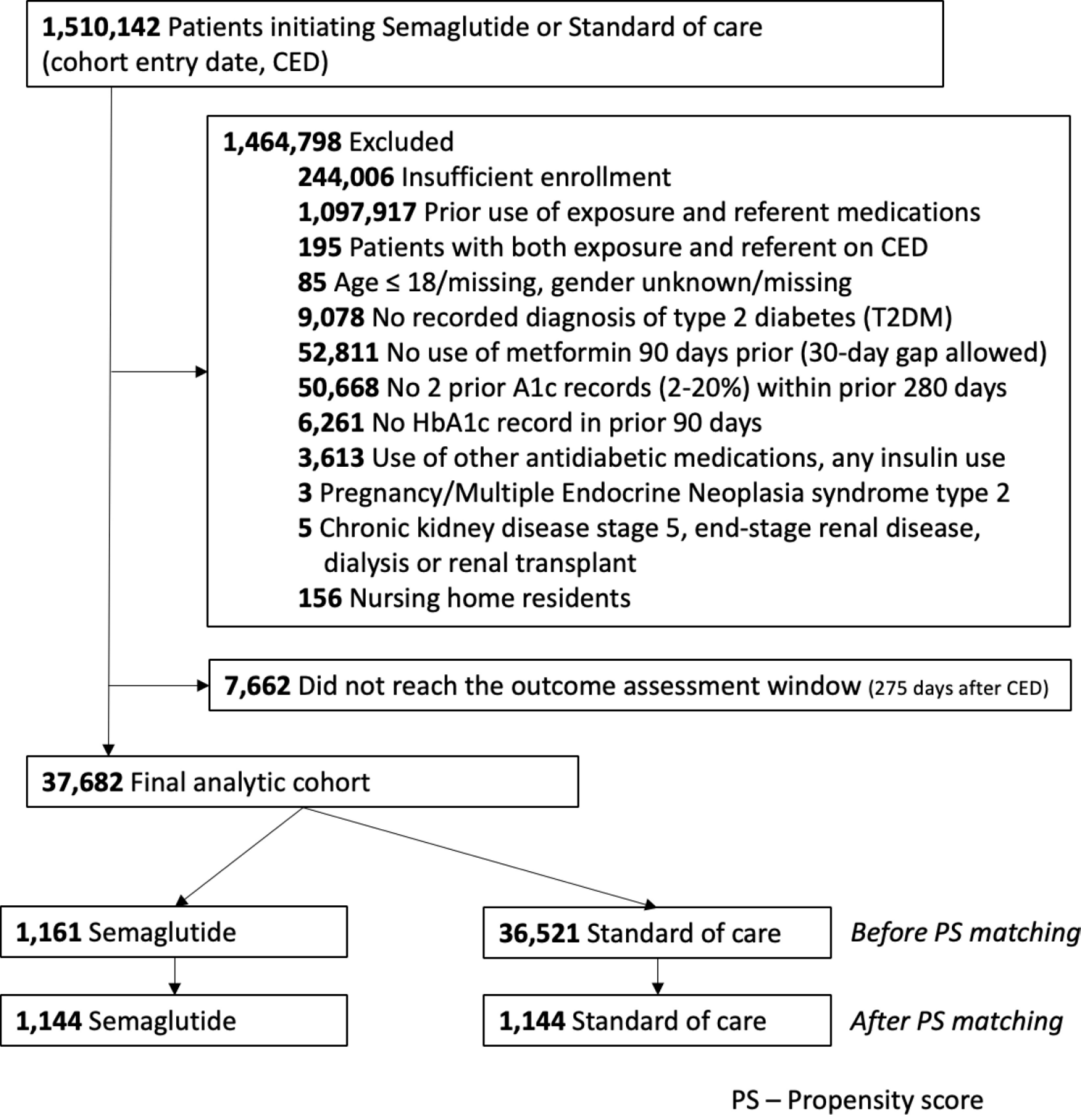
Consolidated Standards of Reporting Trials diagram for the as-started analysis of the primary outcome.

After applying eligibility criteria, we identified 45,344 patients on metformin monotherapy who initiated injectable semaglutide or SoC. Of these, 7,662 (16.9%) did not reach the outcome assessment window for the AS analysis, resulting in a cohort of 37,682 (1161 injectable semaglutide, 36,521 SoC) patients. In the SoC group, there were 19,568 (54.6%) patients prescribed SGLT-2 inhibitors, 6,324 (17.6%) prescribed DPP-4 inhibitors, 6,542 (18.2%) prescribed SUs, and 3,444 (9.6%) prescribed GLP-1RAs (figure 1). Before 1:1 PS matching, semaglutide initiators were younger, more likely to be White, had lower rates of diabetic neuropathy but higher rates of comorbidities such as obesity and mood disorders, and were more likely to be on mood-stabilizing medications. They afforded higher pharmacy copays and were more likely to have commercial insurance. After PS-matching, each group consisted of 1,144 patients, achieving covariate balance with a standardized difference of <0.1 across all variables and a postmatching c-statistic of 0.5 (table 1, online supplemental figure S6). The PS-matched cohort had a mean age of 59 years, with 49% being male and 61% white. Approximately 75% had hypertension, 70% dyslipidemia, and around 40% had obesity recorded (table 1).

**Table 1:** Pre-exposure characteristics of study participants before and after propensity score matching for the as-started analysis of the primary outcome

	Before 1:1 propensity score matching (c=0.8)			After 1:1 propensity score matching (c=0.5)		
	Semaglutide	Standard of care		Semaglutide	Standard of care	
	n=1161	n=36 521	St. Diff	n=1144	n=1144	St. Diff.
Glucose-lowering medications						
Injectable Semaglutide	1161 (100)	0		1144 (100)	0	
SGLT-2i		19 568 (54.6)			533 (47.1)	
DPP-4is		6324 (17.6)			167 (14.8)	
Sulfonylureas		6542 (18.2)			280 (24.8)	
GLP-1RAs (other than injectable semaglutide)		3444 (9.6)			151 (13.3)	
Year of Cohort Entry Date (Calendar time) *, n (%)			0.90			0.04*
December 5, 2017–December 31, 2018	8 (0.7)	10 235 (28.0)		8 (0.7)	77 (6.7)	
January 1, 2019–December 31, 2019	314 (27.0)	10 379 (28.4)		314 (27.4)	205 (17.9)	
January 1, 2020–December 31, 2020	530 (45.7)	10 690 (29.3)		528 (46.2)	506 (44.2)	
1 Jan 2021 – 31 May 2021	309 (26.6)	5217 (14.3)		294 (25.7)	356 (31.1)	
Baseline characteristics						
Age, mean (SD), years	58.58 (11.86)	65.14 (11.41)	0.56	58.74 (11.82)	58.88 (11.42)	0.01
Male, n (%)	568 (48.9)	19 671 (53.9)	0.10	561 (49.0)	562 (49.1)	0.00
White race, n (%)	719 (61.9)	19 379 (53.1)	0.18	706 (61.7)	696 (60.8)	0.02
Burden of comorbidities						
Combined comorbidity score,^24 25^ mean (SD)	2.41 (1.69)	2.64 (1.89)	0.13	2.41 (1.69)	2.40 (1.73)	0.01
Frailty score,^23^ n (%)			0.06			0.05
Robust	320 (27.6)	9161 (25.1)		314 (27.4)	338 (29.5)	
Pre-frail	432 (37.2)	13 672 (37.4)		427 (37.3)	407 (35.6)	
Frail	409 (35.2)	13 688 (37.5)		403 (35.2)	399 (34.9)	
Diabetes-related conditions						
A1C, mean (SD)	8.66 (1.61)	8.61 (1.49)	0.03	8.67 (1.61)	8.73 (1.59)	0.04
Diabetic nephropathy, n (%)	145 (12.5)	6260 (17.1)	0.13	145 (12.7)	139 (12.2)	0.02
Diabetic neuropathy, n (%)	221 (19.0)	7353 (20.1)	0.03	217 (19.0)	216 (18.9)	0.00
Diabetic retinopathy, n (%)	70 (6.0)	2131 (5.8)	0.01	70 (6.1)	68 (5.9)	0.01
Diabetes with unspecified complications, n (%)	89 (7.7)	2122 (5.8)	0.07	88 (7.7)	88 (7.7)	0.00
Diabetes with peripheral circulatory disorders, amputations, and diabetic foot, n (%)	25 (2.2)	578 (1.6)	0.04	24 (2.1)	22 (1.9)	0.01
Cardiovascular comorbidities, n (%)						
Hypertension	269 (23.2)	7066 (19.3)	0.09	862 (75.3)	863 (75.4)	0.00
Hyperlipidemia	807 (69.5)	25 250 (69.1)	0.01	797 (69.7)	785 (68.6)	0.02
Atherosclerosis cardiovascular disease†	192 (16.5)	7437 (20.4)	0.10	188 (16.4)	176 (15.4)	0.03
Cerebrovascular disease‡	24 (2.1)	977 (2.7)	0.04	24 (2.1)	26 (2.3)	0.01
Heart failure	47 (4.0)	2156 (5.9)	0.09	45 (3.9)	51 (4.5)	0.03
Atrial fibrillation	53 (4.6)	2198 (6.0)	0.07	51 (4.5)	52 (4.5)	0.00
Other cardiac dysrhythmia	83 (7.1)	3496 (9.6)	0.09	81 (7.1)	88 (7.7)	0.02
Renal comorbidities, n (%)						
CKD stage 1–2	46 (4.0)	2065 (5.7)	0.08	46 (4.0)	47 (4.1)	0.00
CKD stage 3–4	69 (5.9)	2928 (8.0)	0.08	69 (6.0)	67 (5.9)	0.01
CKD unspecified	21 (1.8)	709 (1.9)	0.01	21 (1.8)	29 (2.5)	0.05
Other comorbidities, n (%)						
Smoking	159 (13.7)	4723 (12.9)	0.02	158 (13.8)	170 (14.9)	0.03
Overweight	84 (7.2)	3723 (10.2)	0.11	83 (7.3)	77 (6.7)	0.02
Obesity	507 (43.7)	10 841 (29.7)	0.29	494 (43.2)	510 (44.6)	0.03
Mood disorders§	175 (15.1)	4781 (13.1)	0.06	171 (14.9)	163 (14.2)	0.02
Obstructive sleep apnea	242 (20.8)	4672 (12.8)	0.22	236 (20.6)	225 (19.7)	0.02
COPD	80 (6.9)	3069 (8.4)	0.06	80 (7.0)	72 (6.3)	0.03
Asthma	82 (7.1)	2052 (5.6)	0.06	81 (7.1)	65 (5.7)	0.06
Osteoarthrosis	186 (16.0)	5356 (14.7)	0.04	184 (16.1)	162 (14.2)	0.05
NASH/NAFLD	82 (7.1)	2005 (5.5)	0.07	80 (7.0)	66 (5.8)	0.05
Other medication use, n (%)						
Antihypertensives¶	923 (79.5)	30 309 (83.0)	0.09	912 (79.7)	916 (80.1)	0.01
Statins or other lipid-lowering drugs**	862 (74.2)	28 165 (77.1)	0.07	853 (74.6)	864 (75.5)	0.02
Opioids	204 (17.6)	5824 (15.9)	0.04	201 (17.6)	194 (17.0)	0.02
Mood stabilizers††	454 (39.1)	10 629 (29.1)	0.21	443 (38.7)	431 (37.7)	0.02
BZDs	104 (9.0)	2994 (8.2)	0.03	103 (9.0)	102 (8.9)	0.00
Gabapentinoids	170 (14.6)	5091 (13.9)	0.02	166 (14.5)	175 (15.3)	0.02
healthcare utilization						
No. of medication claims, mean (SD)	12.28 (7.47)	11.00 (6.97)	0.18	12.19 (7.33)	12.22 (7.86)	0.01
No. of hospitalizations/ED visits, mean (SD)	0.36 (1.07)	0.39 (1.22)	0.02	0.37 (1.08)	0.34 (0.96)	0.02
No. of office visits, mean (SD)	6.09 (4.69)	5.69 (4.74)	0.08	6.06 (4.65)	5.93 (5.11)	0.03
No. of endocrinologist visits, mean (SD)	0.25 (0.72)	0.11 (0.50)	0.23	0.23 (0.65)	0.19 (0.73)	0.06
Brand name prescription, mean (SD)	9.85 (4.84)	9.24 (4.34)	0.13	9.80 (4.79)	9.75 (4.94)	0.01
Generic name prescription, mean (SD)	9.76 (4.77)	9.17 (4.28)	0.13	9.70 (4.72)	9.66 (4.89)	0.01
No. of A1C tests, mean (SD)	1.61 (0.71)	1.57 (0.71)	0.06	1.61 (0.71)	1.58 (0.67)	0.04
Metabolic blood chemistry test, n (%)	938 (80.8)	30 648 (83.9)	0.08	929 (81.2)	928 (81.1)	0.00
Bone density test, n (%)	39 (3.4)	1438 (3.9)	0.03	39 (3.4)	39 (3.4)	0.00
PSA test or prostate exam for DRE, n (%)	219 (18.9)	7147 (19.6)	0.02	216 (18.9)	220 (19.2)	0.01
Flexible sigmoidoscopy, colonoscopy (incl. CT), n (%)	68 (5.9)	1650 (4.5)	0.06	66 (5.8)	65 (5.7)	0.00
Mammograms, n (%)	179 (15.4)	4629 (12.7)	0.08	176 (15.4)	165 (14.4)	0.03
Pap smear, n (%)	60 (5.2)	1112 (3.0)	0.11	58 (5.1)	66 (5.8)	0.03
influenza vaccine, n (%)	231 (19.9)	7137 (19.5)	0.01	226 (19.8)	211 (18.4)	0.03
Pneumococcal vaccine, n (%)	324 (27.9)	10 137 (27.8)	0.00	319 (27.9)	317 (27.7)	0.00
Copay for pharmacy cost, mean (SD),	192.98 (285.35)	157.54 (358.51)	0.11	191.49 (285.22)	165.22 (213.99)	0.10
Insurance type, n (%)			0.44			0.02
Commercial	575 (49.5)	10 408 (28.5)		563 (49.2)	554 (48.4)	
Medicare	586 (50.5)	26 113 (71.5)		581 (50.8)	590 (51.6)	
Low-income indicator, n (%)	122 (10.5)	5235 (14.3)	0.12	120 (10.5)	119 (10.4)	0.00

* The St. Diff. calculated here reflects the value when calendar time is treated as a continuous variable in the propensity score model.

† Defined by Old MI/acute MI/unstable angina/stable angina/other forms of chronic ischemic disease/history of CABG or PTCA/peripheral arterial disease or surgery.

‡ Defined by Stroke/transient ischemic attack/late effects of cerebrovascular disease.

§ Defined by anxiety/depression.

¶ Defined by ACE inhibitors/ARBs/calcium channel blockers/beta blockers/diuretics.

** Defined by statins/other lipid-lowering drugs.

†† Defined by antidepressants/anxiolytics/hypnotics/benzodiazepines.

A1C, glycated hemoglobin; ACE, Angiotensin-converting enzyme; ARBs, Angiotensin II receptor blockers; BZDs, Benzodiazepines; CABG, Coronary artery bypass grafting; CKD, Chronic kidney disease; COPD, Chronic obstructive pulmonary disease; DPP-4is, Dipeptidyl peptidase-4 inhibitors; DRE, Digital rectal examination; GLP-1RAs, Glucagon-like peptide-1 receptor agonists excluding semaglutide injectable; MI, Myocardial infaction; NAFLD, Non-alcoholic fatty liver disease ; NASH, Nonalcoholic steatohepatitis; PSA, Prostate-specific antigen; PTCA, Percutaneous transluminal coronary angioplasty; SGLT-2is, Sodium-glucose cotransporter-2 inhibitors; St. Diff, Standardized difference.

When comparing our matched population to the SEPRA trial cohort, our study population was similar in age, sex, and key comorbidities, such as baseline A1C, dyslipidemia, diabetic neuropathy, myocardial infarction, heart failure, stroke, and thiazide diuretic use. However, our cohort had a higher proportion of non-White patients than the trial and a slightly higher prevalence of diabetic retinopathy, and more use of lipid-lowering medications, beta-blockers, and calcium channel blockers (table 2). Based on the interim results of the SEPRA trial,^14^ 70% of patients completed the study, and the statistical analysis plan indicated that the missing data imputation would be performed for all patients without follow-up A1C measurements, potentially resulting in the imputation of approximately 30% of the values.^14^ In our emulation, 83% of patients reached the outcome assessment window, with 60% having A1C measurements available, imputing the remaining 40%. The degree of missingness in our study is comparable to the SEPRA trial and aligns with patterns observed in other claims-based database studies, where missing A1C values have ranged from 25% to 70%.^1037^,^39^

**Table 2:** Selected patient characteristics from the SEPRA trial and 1:1 propensity score matched database study

	SEPRA trial (n=1278)	Database study (n=2288)	St. diff.*
Patient characteristics			
Age, years, mean (SD)	57.4 (11.1)	58.8 (11.6)	0.12
Male, n (%)	692 (54.2)	1123 (49.1)	0.10
White race, n (%)	1004 (78.6)	1402 (61.3)	0.38
Hypertension, n (%)	986 (77.2)	1725 (75.4)	0.04
Dyslipidemia, n (%)	911 (71.3)	1582 (69.1)	0.05
Diabetic neuropathy, n (%)	179 (14.0)	433 (18.9)	0.13
Diabetic nephropathy, n (%)	50 (3.9)	284 (12.41)	0.32
Diabetic retinopathy, n (%)	25 (2.0)	138 (6.0)	0.21
Myocardial infarction, n (%)	30 (2.3)	54 (2.4)	0.01
Heart failure, n (%)	24 (1.9)	96 (4.2)	0.13
Stroke, n (%)	19 (1.5)	35 (1.5)	0.00
Statins and other lipid-lowering drugs, n (%)	706 (55.5)	1717 (75.0)	0.42
Beta-blockers, n (%)	260 (20.4)	672 (29.6)	0.21
Calcium channel blockers, n (%)	209 (16.4)	576 (25.2)	0.21
Thiazide diuretics, n (%)	168 (13.2)	309 (13.5)	0.01
Baseline A1C (%), mean (SD)	8.5 (1.6)	8.7 (1.6)	0.13
Baseline A1C category, n (%)			0.05
<8	582 (45.5)	973 (43.0)	
≥8.0	696 (54.5)	1315 (57.0)	

* Values <0.1 are considered not meaningful differences in distribution between SEPRA trial and RWD cohort.

A1C, glycated hemoglobin; RWD, real-world-data; SEPRA trial, SEmaglutide PRAgmatic trial; St. Diff, absolute standard difference.

Our study found that patients who initiated treatment with injectable semaglutide were more likely to have achieved glycemic control (A1C <7%) (risk ratio (95% CI), 1.30 (1.16 to 1.45)), than those who initiated SoC medications, compared with interim results observed in the trial (risk ratio (95% CI), 1.16 (0.89 to 1.51)). The 1-year reduction in A1C was 0.2% points in this study compared with 0.4% in the SEPRA trial interim results, whereas the number of severe hypoglycemia events resulting in ED or inpatient visits was similarly low in both studies. Based on the trial’s interim results,^14^ hypoglycemia was reported in <3 participants per arm. We are unable to report exact counts of severe hypoglycemia events in our study due to data use agreements restricting disclosure of cell sizes <11 (table 3). In the trial emulation based on our risk ratio analysis, we achieved concordance on statistical significance and standardized difference but not on estimate agreement. However, in the post-hoc OR analysis, concordance was achieved across all three metrics (table 3). In our secondary—AT analysis, we found that patients treated with injectable semaglutide were more likely to achieve glycemic control than those receiving standard care (risk ratio (95% CI), 1.48 (1.35 to 1.62)). We observed that changes in A1C among patients treated with injectable semaglutide were larger (−1.7% points) than those treated with SoC (−1.2% points) (online supplemental table S4).

**Table 3:** Estimated effects for the prespecified primary outcome of the database analysis compared with the SEPRA trial interim results

	RWD study		SEPRA trial interim results					
	Semaglutide	Standard of care	Semaglutide	Standard of care	Binary agreement metrics			St. Diff
No. PS matched/enrolled patients	n=1144	n=1144	n=644*	n=634*				
No. patients with A1C measured during the outcome assessment window†	697 (60.9%)	687 (60.1%)	430 (66.8%)	462 (72.9%)				
No. primary outcome events (A1C<7.0%)‡	366 (52.5%)	277 (40.3%)	244 (56.7%)	226 (48.9%)				
No. primary outcome events (A1C<7.0%)§	565 (49.4%)	436 (38.1%)						
Follow-up time in days, mean (SD)	377.89 (56.36)	373.69 (54.57)	--	--				
Number needed to treat§	9		--					
Absolute risk difference (95% CI)§	0.11 (0.06 to 0.16)		--					
Risk ratio (95% CI)^§**^	1.30 (1.16 to 1.45)		1.16 (0.89 to 1.51)		**--**	EA	SD	−0.8
OR from logistic regression with baseline A1C included in the model (95% CI)¶	1.56 (1.29 to 1.89)		1.36 (1.03 to 1.79)		SS	EA	SD	−0.8
Change in A1C (baseline to 1-year), mean, (SD) percentage points§	-1.3 (1.9)	-1.1 (1.8)	-1.5	-1.1	n/a	n/a	n/a	n/a
No. of hypoglycemia events over follow-up*^††‡‡^	<11	<11	2	1	n/a	n/a	n/a	n/a

-- = Binary agreement metric was not met

SS = statistical significance agreement: defined by adjusted database study and randomized controlled trial (RCT) estimates and CIs on the same side of null.

EA = estimate agreement: defined by adjusted database study point estimates falling within the 95% CI of the corresponding RCT result.

SD = SD agreement: defined by SDs |Z| < 1.96. SDs are calculated $Z=\frac{{\hat{\Theta }}_{\text{RCT}}-{\hat{\Theta }}_{\text{RWE}}}{\sqrt{{\hat{\sigma }}_{\text{RCT}}^{2}+{\hat{\sigma }}_{\text{RWE}}^{2}}}$, where $\hat{\Theta }$ are the treatment effect estimates (usually log HRs) and the ${\hat{\sigma }}^{2}$ are associated variances. These quantify the difference in effect size between the RCT and database study relative to the pooled SD. Therefore, a SD of 1.00 indicates that the effect estimate from the RCT and the database study are 1 SD apart. Assuming an α-level of 0.05 and assuming that both the database and RCT results are based on large samples, we would reject the null hypothesis of no difference whenever |Z| > 1.96

* 1432 patients in each group were followed from cohort entry date for the number of hypoglycemia outcomes.

† In trial results, this value is the number of people analyzed.

‡ Percentages are based on the number of patients with available A1c values before imputation.

§ Calculated after imputing missing outcomes, steps explained in emethods1.

¶ Post-hoc analysis for the RWD study due to the SEPRA statistical analysis plan becoming available after pre-registration of the RWD study protocol.

** The trial’s unadjusted risk ratio is calculated based on the numbers in the provided 2×2 table.

†† Confidence intervals and/or SD were not available from SEPRA trial so agreement metrics could not be calculated.

‡‡ Cannot report cell counts <11 from RWD where follow-up is from baseline to 1 year; SEPRA counts are from baseline up to 2 years.

A1C, glycated hemoglobin; n/a, not applicable; PS, propensity score; RWD, real-word data; SD, Standard deviation; SEPRA trial, SEmaglutide PRAgmatic trial; SoC, Standard of care medications.

When comparing injectable semaglutide to individual classes of SoC medications (DPP-4is, SGLT-2is, SUs, and GLP-1RAs), the semaglutide group consistently achieved better glycemic control and showed a slightly greater reduction in A1C (online supplemental tables S5a, b). The largest differences in glycemic control were observed when comparing semaglutide to SGLT-2 inhibitors; however, semaglutide also showed modestly greater effectiveness than other GLP-1 receptor agonists. These findings are consistent with prior systematic reviews, meta-analyses, and network meta-analyses demonstrating superior glycemic efficacy of semaglutide within and across drug classes.^40^,^43^

Although computing ORs for frequent endpoints is generally discouraged as it can be a poor approximation of risk ratios,^29 30^ we conducted a post-hoc analysis using OR, resulting in OR (95% CI) of 1.56 (1.29 to 1.89) in our cohort study, compared with OR (95% CI) of 1.36 (1.03 to 1.78) based on the interim results in the SEPRA trial.

All sensitivity analyses and other post-hoc analyses demonstrated a consistent pattern of results, with patients initiating semaglutide being more likely to achieve glycemic control and exhibiting a slightly higher reduction in A1C. The complete case analysis produced a risk ratio (95% CI) of 1.30 (1.16 to 1.46), which also closely aligned with our primary analysis result (table 3, online supplemental tables S6-S9).

## Discussion

### Main findings

This cohort study used longitudinal claims data with lab test results to predict the results of the pragmatic SEPRA trial. Our analyses indicated that patients initiating injectable semaglutide achieved better glycemic control and a greater reduction in A1C compared with those on SoC medications. These findings are comparable with the now-public interim trial findings—with approximately 30% more patients in our study achieving A1C <7% at 1 year after initiating injectable semaglutide compared with SoC. However, both groups were effective at lowering A1C, and we observed that semaglutide reduced A1C by only 0.2% more than SoC, a relatively small improvement that may not translate to large effects on patient complications at a population level.

Due to lower-than-expected recruitment, multiple amendments were made to the SEPRA trial, reducing the original target enrollment of 2,250 by 43% to 1,278, resulting in wider-than-expected CIs. In contrast, our real-world study was able to emulate the original trial question with a sufficiently large sample size, offering a complementary perspective. This adds to the growing body of evidence that the role of well-designed studies using RWD in supporting clinical trials—particularly when trials face challenges such as limited recruitment, protocol amendments,^15^ or early termination.^18^ In such scenarios, RWE can help bridge evidence gaps and provide timely insights into effectiveness and safety in broader patient populations. It also enables the examination of questions related to effectiveness and safety in different populations. Despite our findings aligning with the interim results of the SEPRA trial, we recognize that the final results may differ, and we await the full release of the trial results to assess how closely our predictions match the final results.^14^

### Strengths and limitations

Although we were unable to exactly emulate the final exclusion criteria and analysis plan of the SEPRA trial in our pre-specified protocol (because these details were not public during our protocol development stage), a key strength of this study is the registration of a fully specified protocol, including study design and analytic choices, prior to public release of the trial results. This approach ensures that our design choices are not influenced by knowledge of the trial outcomes. We were also able to clearly describe post-hoc analyses to better align the statistical analyses used in our RWD study with the analyses for the SEPRA trial after the trial’s statistical analysis plan was made public. To our knowledge, this is the first study designed to use RWD to emulate and predict the results of the SEPRA trial. Our study used contemporary statistical methods and generated robust estimates across an array of prespecified sensitivity and subgroup analyses.

Despite employing rigorous analytic methods, residual confounding due to unmeasured factors cannot be entirely ruled out. A major strength of our work lies in the detailed handling of partially observed A1C measurements. Missing data diagnostics suggested that the missingness mechanism likely follows the missing at random assumption,^27^ making multiple imputation a preferred method over complete case analysis. This approach offers several advantages, as it retains all patients in the analysis and provides more realistic SE estimates by accounting for both conventional sampling variance (within-imputation variance) and the additional variance introduced by missing data (between-imputation variance).^44^ Additionally, we conducted multiple sensitivity analyses to demonstrate the robustness of our findings.

As noted earlier, only a subset of patients in our database had laboratory data available, and our inclusion criteria and outcomes were based on the availability of A1C data. However, the availability of this data is driven by lab provider partnerships for Optum Clinformatics. We use our inclusion criteria to balance the availability of labs at baseline, but in routine clinical practice, the frequency and timing of A1C assessments are influenced by various factors, including the type of antidiabetic treatment regimen, A1C level at treatment initiation, the anticipated change in A1C, and adherence to testing requirements.^45^ As a result, limiting our analysis to patients with lab data may impact the generalizability of our findings. In addition, some factors that impact glycemic control, such as lifestyle modifications, duration of diabetes, or body mass index, are not well captured in claims databases and, therefore, could not be accounted for in our analysis. Of these, the duration of diabetes is especially important, given its link to progressive β-cell decline and reduced effectiveness of oral antidiabetic medications over time.^46^ However, the potential bias due to lack of direct measurement of duration of diabetes is mitigated in our study design, which compared initiators of the first second-line therapy started after metformin monotherapy, so patients in both groups likely had a similar duration of diabetes. In the clinical trial, patients paid a maximum of US$20 per month in copay for their medication, with any additional costs reimbursed, regardless of whether they were in the semaglutide or SoC group.^15^ However, in our study, patients were responsible for the full copay, which may vary across insurance plans and thereby contribute to channeling of the patient population as well as differences in adherence. Although we adjusted for proxies of socioeconomic status (eg, race, copay amount, insurance type, low-income indicator that were available in the data^47 48^), there may still be residual confounding from unmeasured factors such as income or health literacy. The direction of this bias is uncertain. Clinicians might preferentially prescribe semaglutide to patients with higher socioeconomic status or who are perceived as more adherent (potentially overestimating its benefit). Alternatively, clinicians might choose to prescribe semaglutide over some alternative SoC therapies for frail older adults of lower socioeconomic status due to concerns regarding adverse effects like hypoglycemia (which would bias the treatment effect toward the null). Additionally, the composition of patients in our study may differ from participants in the SEPRA trial, which may contribute to divergence in results in the presence of effect measure modification. As noted in the results section, there were notable differences regarding baseline characteristics between our routine care population and SEPRA participants. Furthermore, we observed variability in effect sizes observed in subgroup analyses comparing semaglutide to specific classes of drugs contributing to the SoC arm. Additionally, the distribution of glucose-lowering therapies that the SoC arm in the SEPRA trial and the switching patterns over follow-up are not yet publicly available. Differences in such factors between our study and the SEPRA trial could affect the results. However, we note that we observed similar results in our AS analysis that did not censor on treatment switching and our AT analysis, which did censor on switching between arms as well as within class in the SoC arm. Our study includes only patients with commercial insurance in the USA, which may limit the generalizability of these findings to individuals without insurance, those covered by public insurance plans, or populations outside of the USA. Finally, severe hypoglycemia was identified using diagnosis codes from ED visits or inpatient encounters. Although this approach aligns with the SEPRA trial definition, the diagnostic codes used to define severe hypoglycemia have been reported to have a PPV of 70–80%.^24 25^ Any resulting outcome misclassification could be nondifferential, which would bias results toward the null, or differential, creating uncertainty in the direction of potential bias.

## Conclusion

In this cohort study of PS-matched patients with T2DM on metformin monotherapy that was designed to emulate and predict the results of the ongoing SEPRA trial, we found that injectable semaglutide was superior to SoC with 30% more patients achieving A1C <7% at 1 year. Our prediction was consistent with interim results from the trial made available after our analysis was completed. Our findings support the notion that well-designed non-randomized studies using fit-for-purpose data can effectively complement pragmatic RCTs.

## Supplementary material

10.1136/bmjdrc-2025-005180 online supplemental file 1

## Data Availability

Data may be obtained from a third party and are not publicly available. Access to the de-identified source data may be granted to authorized researchers via application and licensing with the data providers: Optum Clinformatics (V8.1, data range from Q1_2004 to Q2_2022), connected@optum.com, https://www.optum.com/business/solutions/life-sciences/real-world-data/claims-data.html.
