# Supplementary material for: Using real-world data to predict findings of an ongoing phase IV trial: glycemic control of semaglutide versus standard of care
Source: BMJ Open Diabetes Res Care. 2025 Oct 29;13(5):e005180. doi: 10.1136/bmjdrc-2025-005180 (PMC12574359; doi:10.1136/bmjdrc-2025-005180)
Supplement: online supplemental file 1 [file bmjdrc-13-5-s001.docx]

**Using Real-world Data to Predict Findings of an Ongoing Phase IV Trial: Glycemic Control of Semaglutide vs Standard of Care**

**Authors:** Sushama Kattinakere Sreedhara^1^, Sebastian Schneeweiss^1^, Elvira D’Andrea^1^, Janick - Weberpals^1^, Elyse C. DiCesare^1^, Elisabetta Patorno^1^, Theodore N. Tsacogianis^1^, Marie C. Bradley ^2^, John Concato^3^, Shirley V. Wang^1^

^1^Division of Pharmacoepidemiology and Pharmacoeconomics, Department of Medicine, Brigham and Women’s Hospital, Harvard Medical School, Boston, MA

^2^Office of Medical Policy, Center for Drug Evaluation and Research, Food and Drug Administration, Silver Spring, MD

^3^Department of Medicine, Yale University School of Medicine, New Haven, CT

Supplementary Materials

Table of contents

1. **Supplement Figures:**
2. Design diagram: As-started cohort.
3. Design diagram: As-Treated cohort.
4. Design diagram: Hypoglycemic episodes cohort.
5. Consort diagram: As-Treated cohort.
6. Consort diagram: Hypoglycemic episodes cohort.
7. K-density plot for propensity score distribution: As-started cohort.
8. K-density plot for propensity score distribution: As-Treated cohort.
9. **Supplement Tables:**
10. Implementation of SEPRA trial eligibility criteria
11. List of all the covariates used in the propensity score model
12. Pre-exposure characteristics of study participants before and after propensity score matching (PSM), As-Treated cohort
13. Results from secondary analysis
14. Results from subgroup analysis
    1. Primary outcome (A1C <7%)
    2. Secondary outcome (change in A1c (baseline to follow-up))
15. Results from sensitivity analysis
    1. Primary outcome (A1C <7%)
    2. Secondary outcome (change in A1C (baseline to follow-up))
16. Results from post hoc analysis, primary outcome (A1C <7%)
17. Results from sensitivity analysis (excluding patients with Medullary carcinoma thyroid (MCT) in the baseline)
18. Results from delta-adjusted Missing Not at Random (MNAR) analysis
19. **Supplement Methods:**
20. Primary analysis: Multiple imputation steps
21. Sensitivity analysis: Multiple imputation steps
22. References

Supplement Figure 1: Design diagram: As-started


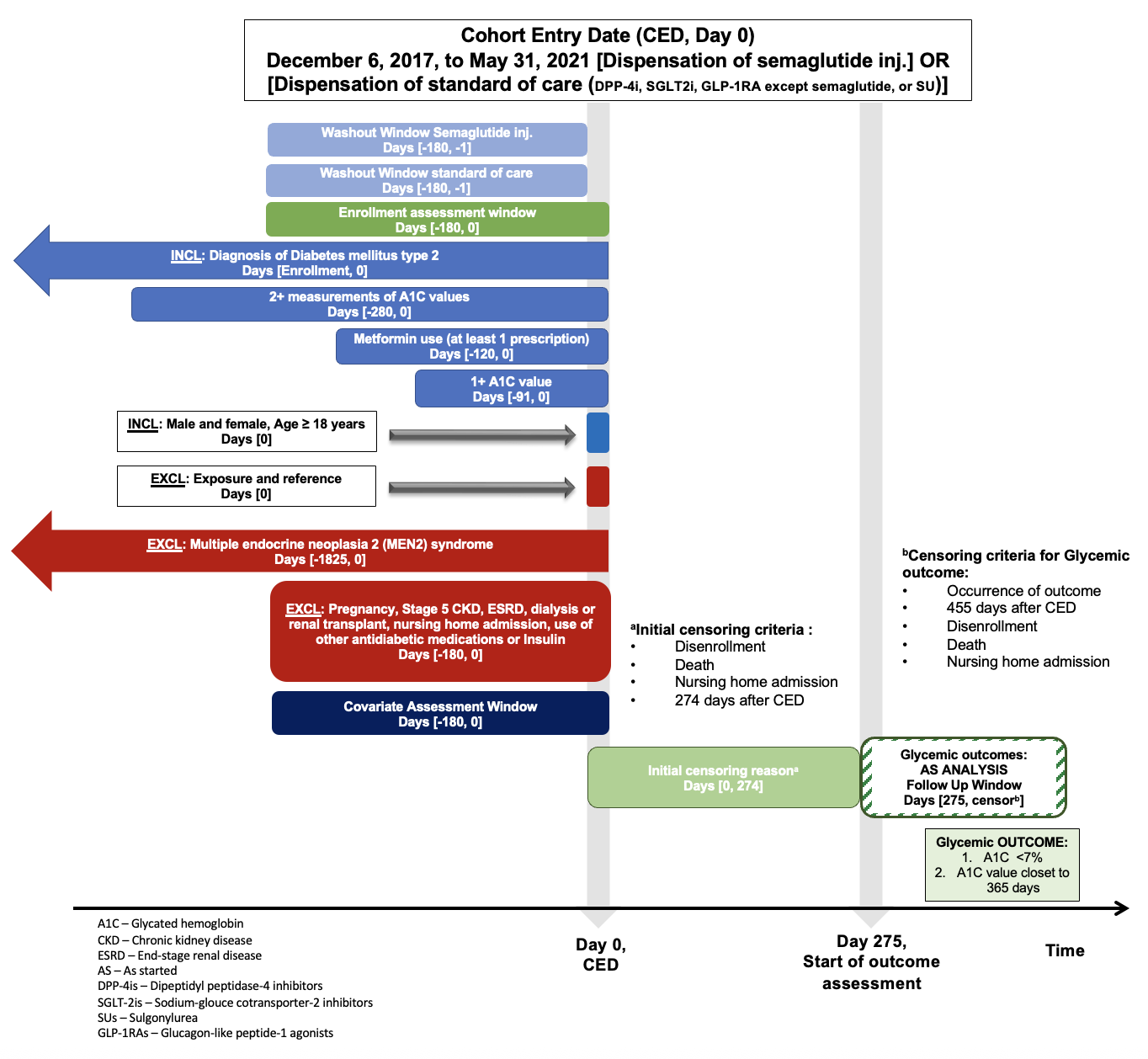


Supplement Figure 2: Design diagram: As-treated(1)


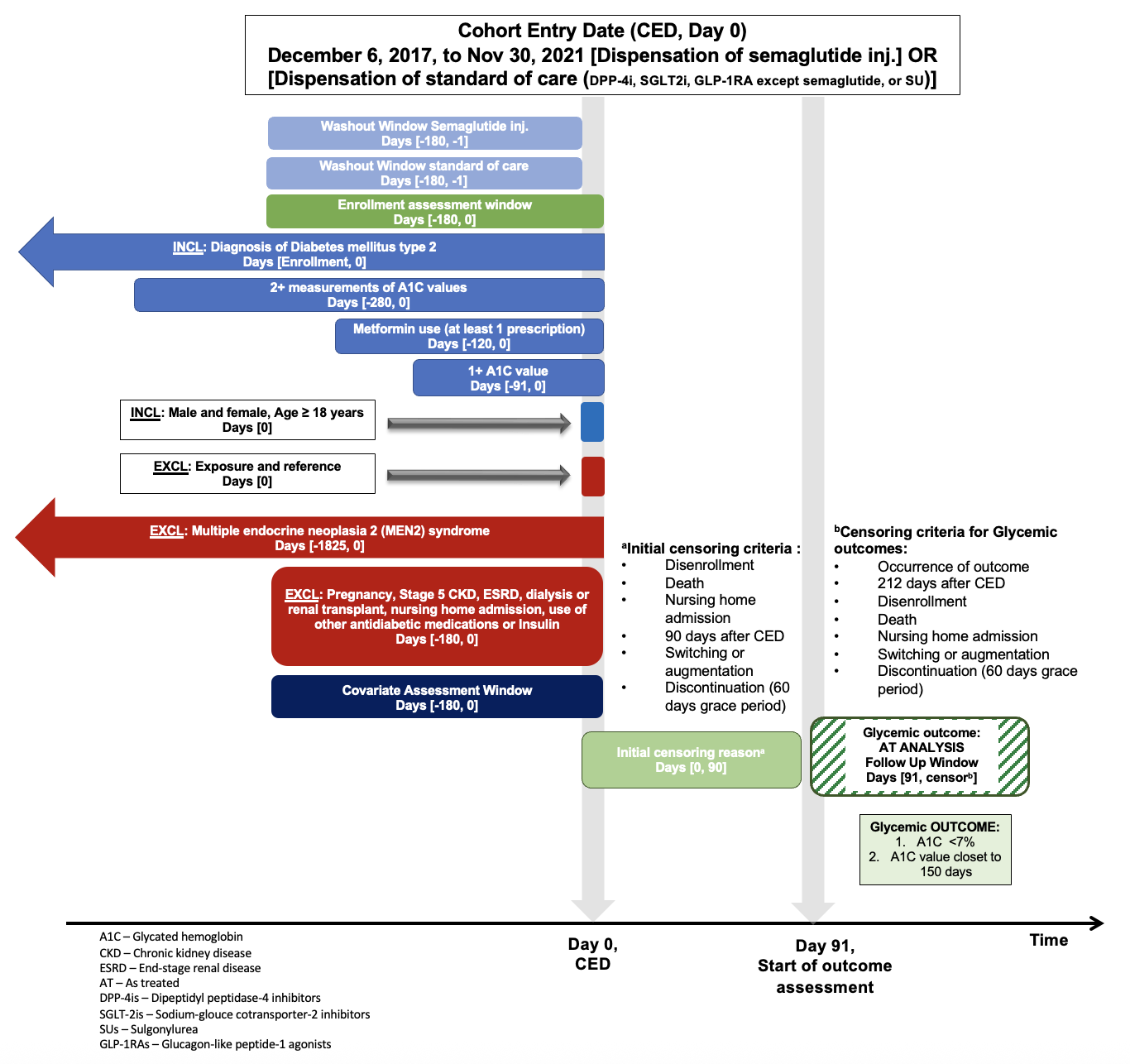


Analogous to the SEPRA trial, and to reflect the effects of semaglutide versus SoC while on treatment, we performed an as-treated (AT) analysis as a secondary analysis. With data available up to May 2022, we extended the cohort entry period to November 30, 2021, to ensure all patients had sufficient time for outcome assessment. Patients had around 150 days of average follow-up time for the AT cohort, and thus, for the AT analysis, the A1C outcomes were assessed as the closest measurement to 150 days (rather than 365 days like AS), allowing for a range of 91 to 212 days to accommodate variation in the timing of outcome assessments.

Supplement Figure 3: Design diagram: Hypoglycemia episodes cohort


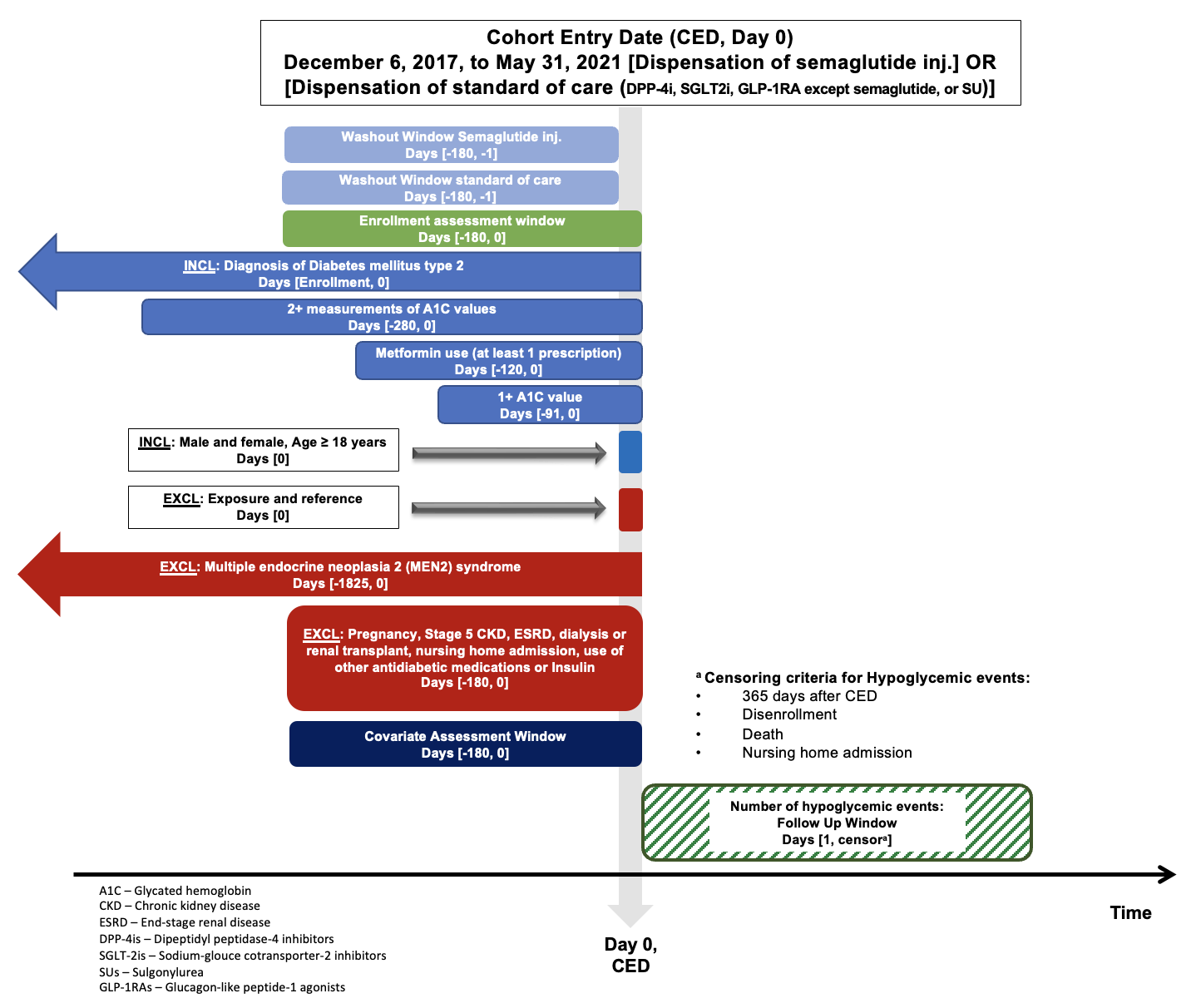


Supplement Figure 4: Consort diagram: As-Treated cohort


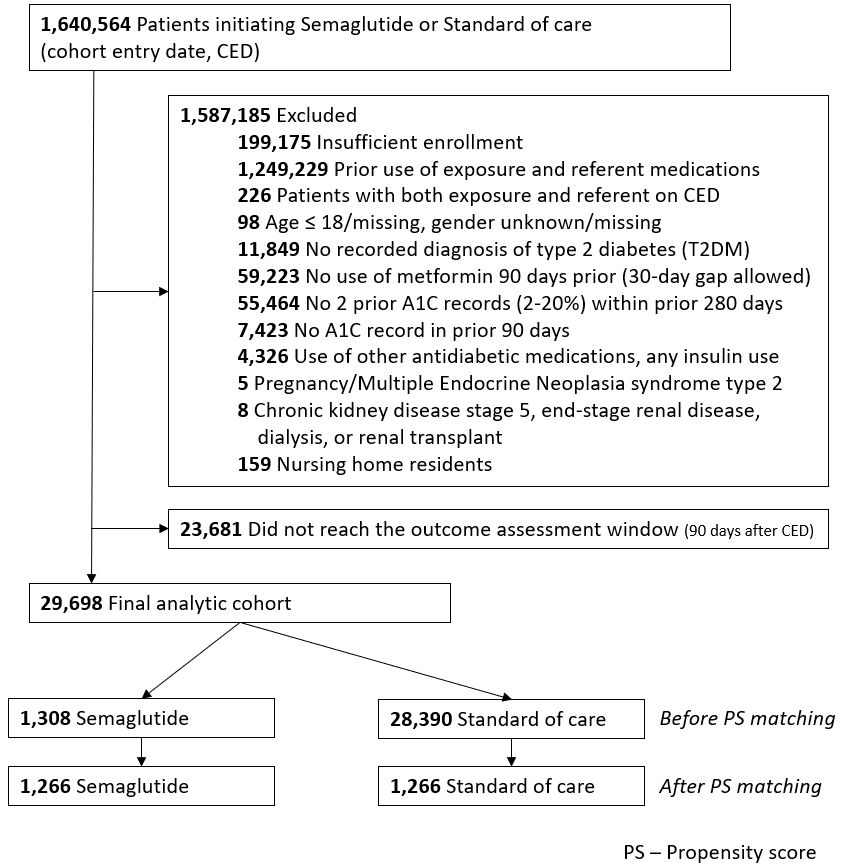


Supplement Figure 5: Consort diagram: Hypoglycemic episode cohort


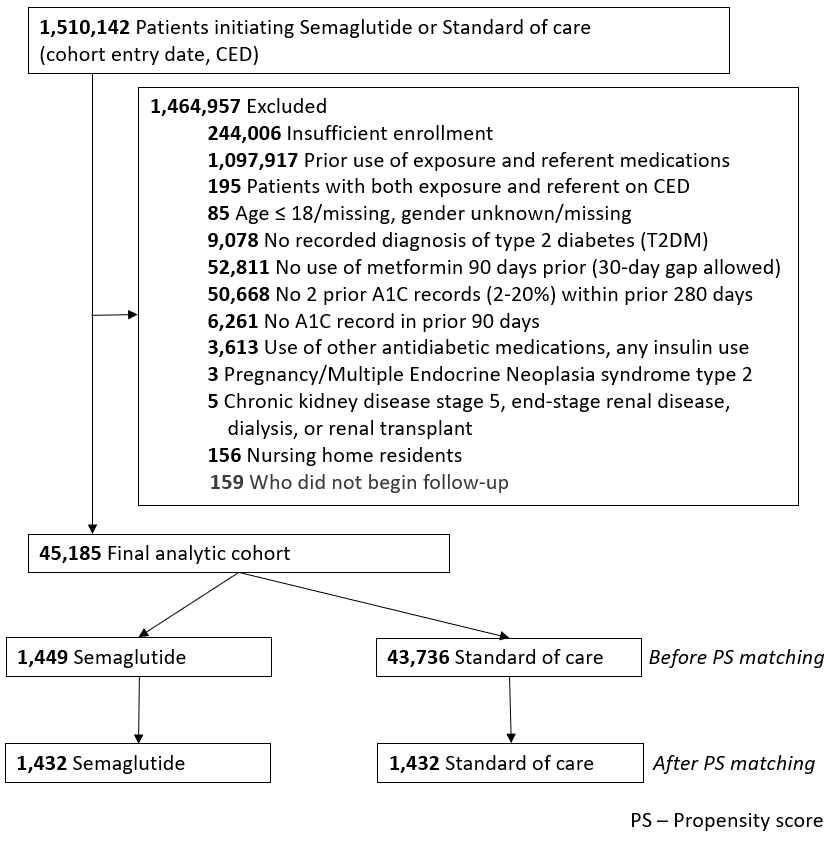


Supplement Figure 6: K-density plot for propensity score distribution: As-started cohort


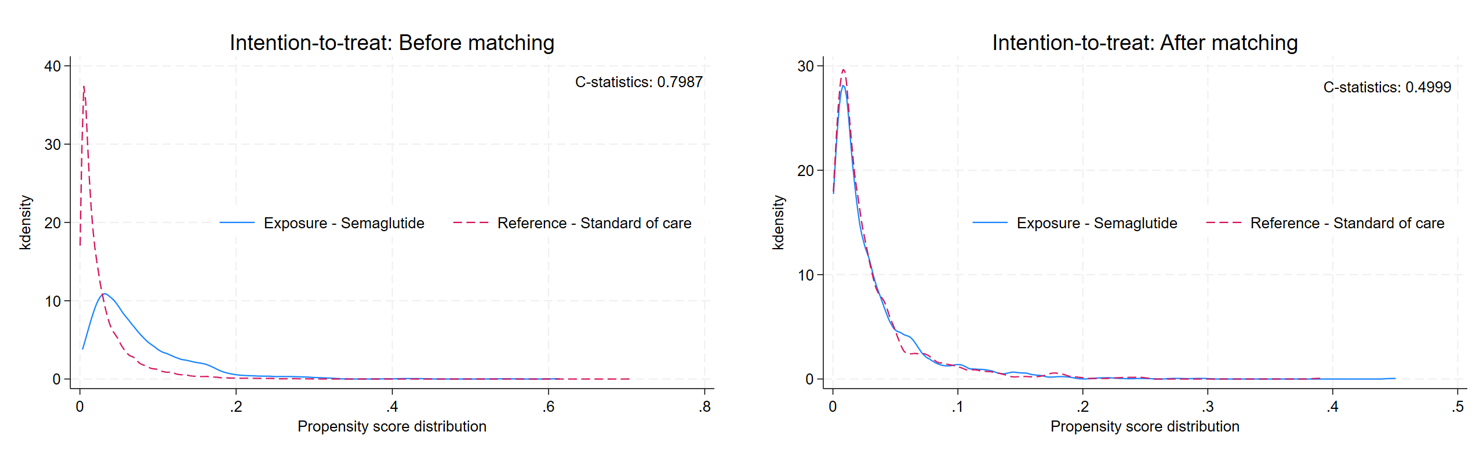


Supplement Figure 7: K-density plot for propensity score distribution: As-Treated cohort


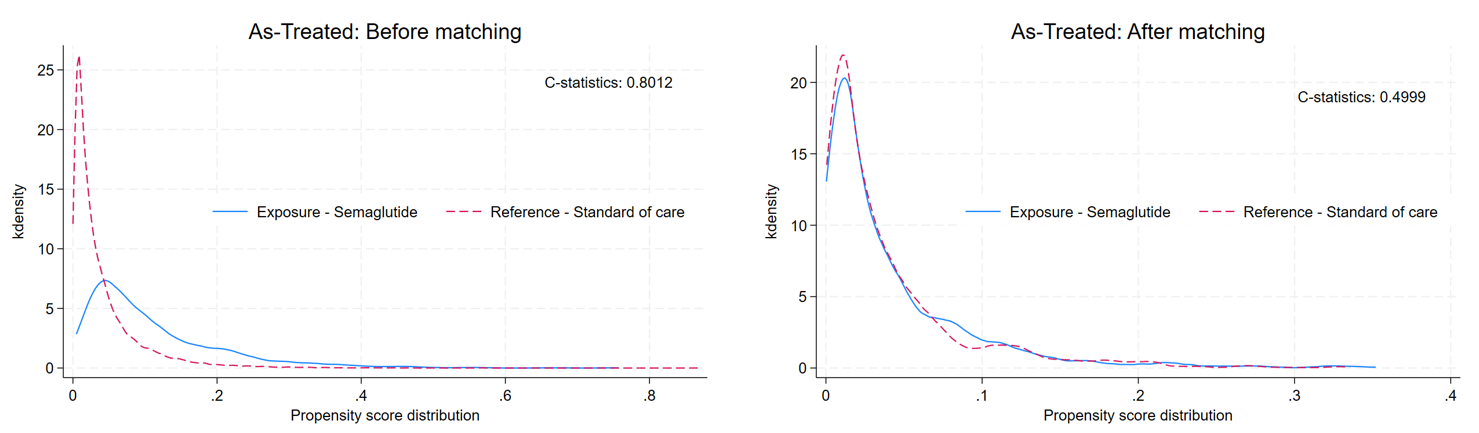


Supplement Table 1. Implementation of SEPRA trial eligibility criteria

| **Study criteria from SEPRA TRIAL(2)** | **Implementation of study criteria from emulation** |
| --- | --- |
| ***Original eligibility criteria (March 2018)*** |  |
| Adult participation (≥18 years) with type 2 diabetes (T2D) | Adult participation (≥18 years) with type 2 diabetes (T2D) |
| Treated with metformin monotherapy | Treated with metformin monotherapy [assessed as metformin dispensation 90 days prior to cohort entry date, with 30 days prescription gap] |
| Requirement for further treatment intensification for glycemic control with an additional antidiabetic medication (treating study physician determined) as per the Food and Drug Administration-approved subcutaneous semaglutide label | Evidence of need for treatment intensification by initiation of semaglutide or standard-of-care medication |
| Current member of an Anthem-affiliated commercial health plan with pharmacy benefits | Continuous enrollment of 180 days, with 30 days allowable gap |
| Recorded glycated hemoglobin value within the last 90 days prior to randomization | At least 2 A1C records (2-20%) within the last 280 days and at least 1 A1C record within the last 90 days prior to the cohort entry date |
| No previous randomization in the study | n/a |
| No treatment with any medication indicated for diabetes other than metformin in the 30 days before the eligibility assessment | No use of anti-diabetes medication other than metformin, including insulin, within the 180 days prior to the cohort entry date |
| No contraindications to semaglutide (as according to the Food and Drug Administration-approved label) | Exclusion for MEN-2 in prior 1,825 days(3)  Exclusion for CKD stage 5, end-stage renal disease, dialysis, or renal transplant in prior 180 days(4) |
| For women, not being pregnant, breastfeeding or intending to become pregnant | Exclusion for pregnancy in prior 180 days to cohort entry date |
| No participation in another clinical trial | n/a |
| ***Amended eligibility criteria (March 2019)*** |  |
| Adult participation (≥18 years) with T2D treated with one or two oral antidiabetic medications | Criterion was not applied because the SEPRA statistical analysis plan, including these amendments, was not publicly available until after the RWD study protocol was pre-registered. |
| ***Amended eligibility criteria (August 2019)*** |  |
| Current member of any health plan with pharmacy benefits | Criterion was not applied because the SEPRA statistical analysis plan, including these amendments, was not publicly available until after the RWD study protocol was pre-registered. |
| ***Amended eligibility criteria (December 2019)*** |  |
| Adult participation (≥18 years) with T2D treated with one or two oral antidiabetic medications, excluding oral semaglutide | Criterion was not applied because the SEPRA statistical analysis plan, including these amendments, was not publicly available until after the RWD study protocol was pre-registered. |
|  | Exclusion for age and gender missing |
|  | Exclusion for nursing home stay in the prior 180 days |

Supplement Table 2. Full list of covariates used in propensity score

| Demographics | Age, sex, race, calendar time |
| --- | --- |
| Burden of comorbidities | Combined comorbidity score (5, 6), frailty score (7) |
| Diabetes-related conditions | A1C value, diabetic nephropathy, diabetic neuropathy, diabetic retinopathy, diabetes with unspecified complications, diabetes with peripheral circulatory disorders, amputations, and diabetic foot |
| Cardiovascular-related comorbidities | Hypertension, hyperlipidemia, atherosclerosis, cardiovascular disease*, cerebrovascular disease^†^, heart failure, atrial fibrillation, other cardiac dysrhythmia |
| Renal comorbidities | CKD stage 1-2, CKD stage 3-4, CKD unspecified |
| Other comorbidities | Smoking, overweight, obesity, mood disorders^‡^, obstructive sleep apnea, COPD, asthma, osteoarthritis, NASH/NAFLD |
| Other medication use | Antihypertensives^§^, statins or other lipid-lowering drugs^\|\|^, opioids, mood stabilizers^¶^, BZDs, gabapetinoids |
| Health-care utilization | Number of medication claims, number of hospitalizations/ED visits/ office visits/endocrinologist visits, brand name prescription, generic name prescription, number of A1C tests, basic or comprehensive metabolic chemistry test, bone density test, PSA test or prostate exam for DRE, flexible sigmoidoscopy, colonoscopy (including CT), mammograms, pap smear, flu vaccine, pneumococcal vaccine, copay for pharmacy cost, insurance type, low-income indicator |
| * Defined by Old MI/Acute MI/Unstable Angina/Stable Angina/Other forms of chronic ischemic disease/History of CABG or PTCA/Peripheral arterial disease or surgery  ^†^ Defined by Stroke/transient ischemic attack/Late effects of cerebrovascular disease  ^‡^ Defined by Anxiety/Depression  ^§^ Defined by ACE inhibitors/ARBs/Calcium channel blockers/Beta blockers/Diuretics  ^\|\|^ Defined by Statins/other lipid-lowering drugs  ^¶^ Defined by Antidepressants/Anxiolytics/Hypnotics/Benzodiazepines  A1C, Glycated hemoglobin; MI; Myocardial infaction; CABG, Coronary artery bypass grafting; PTCA, Percutaneous Transluminal Coronary Angioplasty; CKD, Chronic kideny disease; COPD, Chronic obstructive pulmonary disease; NASH, Nonalcoholic steatohepatitis; NAFLD, nonalcoholic fatty liver disease; ACE, Angiotensin-converting enzyme; ARBs; Angiotensin II receptor blockers; BZDs, Benzodiazepines; PSA, Prostate-specific antigen; DRE,Digital Rectal Examination | |

| Supplement Table 3. Pre-exposure characteristics of study participants before and after propensity score matching (PSM) for the as-started analysis of the primary outcome | | | | | | |
| --- | --- | --- | --- | --- | --- | --- |
|  | Before 1:1 Propensity score matching (c= 0.8) | |  | After 1:1 Propensity score matching (c= 0.5) | |  |
|  | Semaglutide | Standard of care |  | Semaglutide | Standard of care |  |
|  | *n = 1,308* | *n = 28,390* | St.Diff | *n = 1,266* | *n = 1,266* | St. Diff. |
| Glucose-lowering medications |  |  |  |  |  |  |
| Injectable Semaglutide | 1,308 (100) | 0 |  | 1,266 (100) | 0 |  |
| SGLT-2i |  | 5,109 (18.1) |  |  | 327 (25.9) |  |
| DPP-4is |  | 4,222 (15.0) |  |  | 170 (13.5) |  |
| SUs |  | 16,720 (59.2) |  |  | 608 (48.2) |  |
| GLP-1Ras (other than injectable semaglutide) |  | 2,177 (7.7) |  |  | 156 (12.4) |  |
|  |  |  |  |  |  |  |
| Year of Cohort Entry Date (Calendar time), n (%) |  |  | 0.84 |  |  | 0.04^#^ |
| 5 Dec 2017 – 31 Dec 2018 | 5 (0.4) | 6,697 (23.6) |  | 5 (0.4) | 61 (4.8) |  |
| 1 Jan 2019 – 31 Dec 2019 | 252 (19.3) | 6,849 (24.1) |  | 251 (19.8) | 159 (12.6) |  |
| 1 Jan 2020 – 31 Dec 2020 | 406 (31.0) | 7,226 (25.5) |  | 401 (31.7) | 370 (29.2) |  |
| 1 Jan 2021 – 31 May 2021 | 645 (49.3) | 7,618 (26.8) |  | 609 (48.1) | 676 (53.4) |  |
| Baseline characteristics |  |  |  |  |  |  |
| Age, mean (SD), years | 58.56 (12.01) | 65.30 (11.29) | 0.58 | 58.91 (11.94) | 59.89 (11.52) | 0.08 |
| Male, n (%) | 608 (46.5) | 15,751 (55.5) | 0.18 | 598 (47.2) | 580 (45.8) | 0.03 |
| White race, n (%) | 858 (65.6) | 15,590 (54.9) | 0.22 | 823 (65.0) | 828 (65.4) | 0.01 |
| Burden of comorbidities |  |  |  |  |  |  |
| Combined comorbidity score(5, 6), mean (SD) | 2.27 (1.59) | 2.57 (1.86) | 0.18 | 2.26 (1.59) | 2.28 (1.64) | 0.01 |
| Frailty score(7), n (%) |  |  | 0.06 |  |  | 0.04 |
| Robust | 366 (28.0) | 7,481 (26.4) |  | 351 (27.7) | 367 (29.0) |  |
| Pre-frail | 506 (38.7) | 10,683 (37.6) |  | 494 (39.0) | 471 (37.2) |  |
| Frail | 436 (33.3) | 10,226 (36.0) |  | 421 (33.3) | 428 (33.8) |  |
| Diabetes-related conditions |  |  |  |  |  |  |
| A1C, mean (sd) | 8.50 (1.48) | 8.53 (1.43) | 0.03 | 8.52 (1.49) | 8.45 (1.31) | 0.05 |
| Diabetic nephropathy, n (%) | 164 (12.5) | 4,476 (15.8) | 0.09 | 158 (12.5) | 162 (12.8) | 0.01 |
| Diabetic neuropathy, n (%) | 222 (17.0) | 5,252 (18.5) | 0.04 | 217 (17.1) | 223 (17.6) | 0.01 |
| Diabetic retinopathy, n (%) | 50 (3.8) | 1,412 (5.0) | 0.06 | 50 (3.9) | 52 (4.1) | 0.01 |
| Diabetes with unspecified complications, n (%) | 88 (6.7) | 1,550 (5.5) | 0.05 | 85 (6.7) | 86 (6.8) | 0.00 |
| Diabetes with peripheral circulatory disorders, amputations, and diabetic foot, n (%) | 21 (1.6) | 465 (1.6) | 0.00 | 21 (1.7) | 17 (1.3) | 0.03 |
| Cardiovascular comorbidities, n (%) |  |  |  |  |  |  |
| Hypertension | 294 (22.5) | 5,912 (20.8) | 0.04 | 941 (74.3) | 962 (76.0) | 0.04 |
| Hyperlipidemia | 896 (68.5) | 19,550 (68.9) | 0.01 | 870 (68.7) | 857 (67.7) | 0.02 |
| Atherosclerosis cardiovascular disease * | 183 (14.0) | 5,612 (19.8) | 0.16 | 183 (14.5) | 174 (13.7) | 0.02 |
| Cerebrovascular disease ^†^ | 27 (2.1) | 768 (2.7) | 0.04 | 27 (2.1) | 24 (1.9) | 0.02 |
| Heart failure | 45 (3.4) | 1630 (5.7) | 0.11 | 43 (3.4) | 41 (3.2) | 0.01 |
| Atrial fibrillation | 56 (4.3) | 1730 (6.1) | 0.08 | 55 (4.3) | 50 (3.9) | 0.02 |
| Other cardiac dysrhythmia | 92 (7.0) | 2711 (9.5) | 0.09 | 90 (7.1) | 87 (6.9) | 0.01 |
| Renal comorbidities, n (%) |  |  |  |  |  |  |
| CKD stage 1-2 | 49 (3.7) | 1,448 (5.1) | 0.07 | 47 (3.7) | 51 (4.0) | 0.02 |
| CKD stage 3-4 | 51 (3.9) | 1,817 (6.4) | 0.11 | 51 (4.0) | 52 (4.1) | 0.00 |
| CKD unspecified | 17 (1.3) | 504 (1.8) | 0.04 | 17 (1.3) | 13 (1.0) | 0.03 |
| Other comorbidities, n (%) |  |  |  |  |  |  |
| Smoking | 169 (12.9) | 3,707 (13.1) | 0.00 | 165 (13.0) | 162 (12.8) | 0.01 |
| Overweight | 85 (6.5) | 2,741 (9.7) | 0.12 | 82 (6.5) | 77 (6.1) | 0.02 |
| Obesity | 582 (44.5) | 8,376 (29.5) | 0.31 | 548 (43.3) | 580 (45.8) | 0.05 |
| Mood disorders ^‡^ | 211 (16.1) | 3,682 (13.0) | 0.09 | 203 (16.0) | 182 (14.4) | 0.05 |
| Obstructive sleep apnea | 280 (21.4) | 3,671 (12.9) | 0.23 | 261 (20.6) | 282 (22.3) | 0.04 |
| COPD | 95 (7.3) | 2,364 (8.3) | 0.04 | 90 (7.1) | 94 (7.4) | 0.01 |
| Asthma | 97 (7.4) | 1,536 (5.4) | 0.08 | 90 (7.1) | 110 (8.7) | 0.06 |
| Osteoarthrosis | 197 (15.1) | 4,096 (14.4) | 0.02 | 191 (15.1) | 208 (16.4) | 0.04 |
| NASH/NAFLD | 86 (6.6) | 1,542 (5.4) | 0.05 | 81 (6.4) | 87 (6.9) | 0.02 |
| Other medication use, n (%) |  |  |  |  |  |  |
| Antihypertensives ^§^ | 1,048 (80.1) | 23,719 (83.5) | 0.09 | 1022 (80.7) | 1032 (81.5) | 0.02 |
| Statins or other lipid-lowering drugs ^\|\|^ | 978 (74.8) | 22,474 (79.2) | 0.10 | 956 (75.5) | 1002 (79.1) | 0.09 |
| Opioids | 210 (16.1) | 4,297 (15.1) | 0.03 | 201 (15.9) | 201 (15.9) | 0.00 |
| Mood stabilizers ^¶^ | 503 (38.5) | 8,216 (28.9) | 0.20 | 480 (37.9) | 487 (38.5) | 0.01 |
| BZDs | 113 (8.6) | 2,245 (7.9) | 0.03 | 111 (8.8) | 112 (8.8) | 0.00 |
| Gabapentinoids | 194 (14.8) | 3,843 (13.5) | 0.04 | 187 (14.8) | 210 (16.6) | 0.05 |
| Health care utilization |  |  |  |  |  |  |
| No. of medication claims, mean (sd) | 12.31 (7.34) | 11.11 (6.97) | 0.17 | 12.18 (7.19) | 12.31 (7.45) | 0.02 |
| No. of hospitalizations/ED visit, mean (sd) | 0.32 (1.14) | 0.37 (1.29) | 0.04 | 0.33 (1.16) | 0.32 (1.03) | 0.01 |
| No. of office visits, mean (sd) | 5.94 (4.65) | 5.57 (4.73) | 0.08 | 5.87 (4.61) | 6.08 (5.18) | 0.04 |
| No. of endocrinologist visits, mean (sd) | 0.24 (0.67) | 0.09 (0.45) | 0.27 | 0.21 (0.59) | 0.17 (0.62) | 0.07 |
| Brand name prescription, mean (sd) | 9.66 (4.65) | 9.19 (4.30) | 0.11 | 9.58 (4.58) | 9.88 (4.80) | 0.06 |
| Generic name prescription, mean (sd) | 9.55 (4.56) | 9.11 (4.25) | 0.10 | 9.48 (4.49) | 9.78 (4.71) | 0.07 |
| No. of A1C test, mean (sd) | 1.60 (0.69) | 1.57 (0.70) | 0.05 | 1.60 (0.69) | 1.61 (0.67) | 0.01 |
| Basic or comprehensive metabolic blood chemistry test, n (%) | 1,063 (81.3) | 23,904 (84.2%) | 0.08 | 1032 (81.5) | 1031 (81.4) | 0.00 |
| Bone density test, n (%) | 48 (3.7) | 1,139 (4.0) | 0.02 | 46 (3.6) | 51 (4.0) | 0.02 |
| PSA test or prostate exam for DRE, n (%) | 228 (17.4) | 5,746 (20.2) | 0.07 | 226 (17.9) | 232 (18.3) | 0.01 |
| Flexible sigmoidoscopy, colonoscopy, (incl. CT), n (%) | 75 (5.7) | 1,365 (4.8) | 0.04 | 70 (5.5) | 69 (5.5) | 0.00 |
| Mammograms, n (%) | 194 (14.8) | 3,545 (12.5) | 0.07 | 186 (14.7) | 202 (16.0) | 0.04 |
| Pap smear, n (%) | 64 (4.9) | 846 (3.0) | 0.10 | 61 (4.8) | 60 (4.7) | 0.00 |
| Flu vaccine, n (%) | 236 (18.0) | 5,159 (18.2) | 0.00 | 227 (17.9) | 221 (17.5) | 0.01 |
| Pneumococcal vaccine, n (%) | 372 (28.4) | 7,679 (27.0) | 0.03 | 360 (28.4) | 365 (28.8) | 0.01 |
| Copay for pharmacy cost, mean (sd), $ | 186.94 (307.18) | 156.34 (303.34) | 0.10 | 181.26 (258.41) | 182.35 (315.15) | 0.00 |
| Insurance type, n (%) |  |  | 0.48 |  |  | 0.08 |
| Commercial | 663 (50.7) | 7,919 (27.9) |  | 628 (49.6) | 577 (45.6) |  |
| Medicare | 645 (49.3) | 20,471 (72.1) |  | 638 (50.4) | 689 (54.4) |  |
| Low-income indicator, n (%) | 133 (10.2) | 4,211 (14.8) | 0.14 | 130 (10.3) | 140 (11.1) | 0.03 |
| * Defined by Old MI/Acute MI/Unstable Angina/Stable Angina/Other forms of chronic ischemic disease/History of CABG or PTCA/Peripheral arterial disease or surgery  ^†^ Defined by Stroke/transient ischemic attack/Late effects of cerebrovascular disease  ^‡^ Defined by Anxiety/Depression  ^§^ Defined by ACE inhibitors/ARBs/Calcium channel blockers/Beta blockers/Diuretics  ^\|\|^ Defined by Statins/other lipid-lowering drugs  ^¶^ Defined by Antidepressants/Anxiolytics/Hypnotics/Benzodiazepines  ^#^ The St. Diff. (standardized difference) calculated here reflects the value when calendar time is treated as a continuous variable in the propensity score model  St. Diff, Standardized difference; DPP-4is, Dipeptidyl peptidase-4 inhibitors; SGLT-2is, Sodium-glucose Cotransporter-2 Inhibitors;  SUs, Sulfonylurea (SUs); GLP-1Ras, Glucagon-like peptide-1 agonists excluding semaglutide injectable; A1C, Glycated hemoglobin; MI; Myocardial infaction; CABG, Coronary artery bypass grafting; PTCA, Percutaneous Transluminal Coronary Angioplasty; CKD, Chronic kideny disease; COPD, Chronic obstructive pulmonary disease; NASH, Nonalcoholic steatohepatitis; NAFLD, nonalcoholic fatty liver disease; ACE, Angiotensin-converting enzyme; ARBs; Angiotensin II receptor blockers; BZDs, Benzodiazepines; PSA, Prostate-specific antigen; DRE,Digital Rectal Examination | | | | | | |

| Supplement Table 4: Results from secondary analysis | | |
| --- | --- | --- |
|  | AT analysis | |
|  | Semaglutide | Standard of care |
|  | *n = 1,266* | *n = 1,266* |
| No. patients with A1C during the outcome assessment window | 589 (46.5%) | 624 (49.3%) |
| No. primary outcome events (A1C < 7.0%) | 386 (65.5%) | 285 (45.7%) |
| Risk ratio*^†^ | 1.48 (1.35, 1.62) | |
| Odds ratio after adjusting for baseline A1C*^†^ | 2.42 (2.01, 2.93) | |
| Change in A1C (baseline to follow-up), mean, (standard deviation)* | -1.7 (1.7) | -1.2 (1.6) |
| No., Number of; Semaglutide, Injectable Semaglutide; SoC, Standard of care medications; AT, as-treated  * The AT analysis results are from the propensity-score-matched imputed dataset, steps explained in supplement methods1  ^†^ Calculated from the marginal 2x2 table after imputing missing outcomes | | |

| Supplement Table 5a: Results from subgroup analysis, primary outcome (A1C <7%) | | |
| --- | --- | --- |
|  | AS analysis | AT analysis |
| Primary Outcome | Risk ratio* | |
| Semaglutide vs SoC, DPP4-is | 1.36 (1.21, 1.53) | 1.59 (1.43, 1.76) |
| Semaglutide vs SoC, SGLT-2is | 1.70 (1.50, 1.93) | 1.71 (1.55, 1.89) |
| Semaglutide vs SoC, SUs | 1.36 (1.21, 1.51) | 1.49 (1.36, 1.64) |
| Semaglutide vs SoC, GLP-1RAs | 1.32 (1.19, 1.47) | 1.13 (1.05, 1.23) |
| AS, As-started; AT, As-treated, Semaglutide, Injectable Semaglutide; SoC, Standard of care medications  DPP-4is, Dipeptidyl peptidase-4 inhibitors; SGLT-2is, Sodium-glucose Cotransporter-2 Inhibitors;  SUs, Sulfonylurea (SUs); GLP-1Ras, Glucagon-like peptide-1 agonists excluding semaglutide injectable  *The AS and AT analysis results are from the propensity-score-matched imputed dataset, steps explained in supplement methods1 | | |

| Supplement Table 5b: Results from subgroup analysis, secondary outcome (change in A1C: baseline to follow-up) | | | | |
| --- | --- | --- | --- | --- |
|  | AS analysis | | AT analysis | |
|  | Semaglutide | SoC | Semaglutide | SoC |
| Secondary Outcome | Mean, SD, Median* | | | |
| Semaglutide vs SoC, DPP4-is | -1.4, 1.9, -1.3 | -1.0, 1.9, -0.9 | -1.7, 1.7, -1.4 | -1.1, 1.6, -0.9 |
| Semaglutide vs SoC, SGLT-2is | -1.4, 2.0, -1.3 | -0.9, 1.8, -0.8 | -1.7, 1.7, -1.4 | -1.2, 1.6, -0.9 |
| Semaglutide vs SoC, SUs | -1.4, 2.0, -1.3 | -1.2, 1.7, -0.9 | -1.8, 1.7, -1.4 | -1.2, 1.7, -1.0 |
| Semaglutide vs SoC, GLP-1RAs | -1.4, 2.0, -1.3 | -1.1, 1.8, -1.0 | -1.8, 1.7, -1.5 | -1.6, 1.6, -1.3 |
| AS, As-started; AT, As-treated, Semaglutide, Injectable Semaglutide; SoC, Standard of care medications  DPP-4is, Dipeptidyl peptidase-4 inhibitors; SGLT-2is, Sodium-glucose Cotransporter-2 Inhibitors;  SUs, Sulfonylurea (SUs); GLP-1Ras, Glucagon-like peptide-1 agonists excluding semaglutide injectable  *The AS and AT analysis results are from the propensity-score-matched imputed dataset, steps explained in supplement methods1 | | | | |

| Supplement Table 6a: Results from sensitivity analysis, primary outcome (A1C <7%) | | |
| --- | --- | --- |
|  | AS analysis | AT analysis |
| Primary Outcome | Risk ratio* | |
| Complete case analysis, unmatched cohort | 1.49 (1.39, 1.61) | 1.52 (1.43, 1.61) |
| Complete case analysis, matched cohort | 1.30 (1.16, 1.46) | 1.43 (1.29, 1.59) |
| Multiple imputation, matched cohort* | 1.31 (1.26, 1.35) | 1.44 (1.39, 1.49) |
| Tipping point analysis (overall), matched cohort^#^ |  |  |
| Mean | 1.32 (1.16, 1.51) | 1.35 (1.19, 1.55) |
| Median | 1.32 (1.16, 1.51) | 1.15 (1.10, 1.19) |
| 1^st^ percentile | 1.11 (1.05, 1.17) | 1.15 (1.10, 1.19) |
| 10^th^ percentile | 1.11 (1.05, 1.17) | 1.15 (1.10, 1.19) |
| 25^th^ percentile | 1.11 (1.05, 1.17) | 1.15 (1.10, 1.19) |
| 75^th^ percentile | 1.32 (1.16, 1.51) | 1.35 (1.19, 1.54) |
| 90^th^ percentile | 1.32 (1.16, 1.51) | 1.35 (1.19, 1.54) |
| 99^th^ percentile | 1.32 (1.16, 1.51) | 1.35 (1.19, 1.54) |
| Tipping point analysis (by exposure), matched cohort^#^ |  |  |
| Mean | 1.32 (1.16, 1.51) | 3.73 (3.36, 4.14) |
| Median | 2.94 (2.63, 3.27) | 3.73 (3.36, 4.14) |
| 1^st^ percentile | 1.11 (1.05, 1.17) | 1.15 (1.10, 1.19) |
| 10^th^ percentile | 1.11 (1.05, 1.17) | 1.15 (1.10, 1.19) |
| 25^th^ percentile | 1.11 (1.05, 1.17) | 1.15 (1.10, 1.19) |
| 75^th^ percentile | 1.32 (1.16, 1.51) | 1.35 (1.19, 1.54) |
| 90^th^ percentile | 1.32 (1.16, 1.51) | 1.35 (1.19, 1.54) |
| 99^th^ percentile | 1.32 (1.16, 1.51) | 1.35 (1.19, 1.54) |
| AS, As-started; AT, As-treated, Semaglutide, Injectable Semaglutide; SoC, Standard of care medications  *Steps explained in Supplement methods 2 | | |

| Supplement Table 6b: Results from sensitivity analysis, secondary outcome (change in A1C: baseline to follow-up) | | | | |
| --- | --- | --- | --- | --- |
|  | AS analysis | | AT analysis | |
|  | Semaglutide | SoC | Semaglutide | SoC |
| Secondary Outcome | Mean, SD, Median* | | | |
| Complete case analysis, unmatched cohort | -1.3, 1.9, -1.2 | -1.1, 1.8, -0.9 | -1.7, 1.7, -1.4 | -1.2, 1.6, -1.0 |
| Complete case analysis, matched cohort | -1.3, 1.9, -1.2 | -0.9, 1.6, -0.8 | -1.7, 1.5, -1.4 | -1.1, 1.4, -0.9 |
| Multiple imputation, matched cohort* | -1.4, 1.9, -1.3 | -1.1, 1.8, -0.9 | -1.7, 1.6, -1.5 | -1.2, 1.5, -1.0 |
| AS, As-started; AT, As-treated, Semaglutide, Injectable Semaglutide; SoC, Standard of care medications  *Steps explained in Supplement methods 2 | | | | |

| Supplement Table 7: Results from post hoc analysis, primary outcome (A1C <7%) | | |
| --- | --- | --- |
|  | AS analysis | AT analysis |
|  | Adjusted Odds ratio* | |
| Multiple imputation, full cohort | 1.56 (1.29, 1.89) | 2.42 (2.01, 2.93) |
| Multiple imputation, subgroup |  |  |
| Semaglutide vs SoC, DPP4-is | 1.77 (1.44, 2.18) | 2.71 (2.19, 3.36) |
| Semaglutide vs SoC, SGLT-2is | 2.46 (2.00, 3.03) | 3.10 (2.54, 3.79) |
| Semaglutide vs SoC, SUs | 1.75 (1.44, 2.13) | 2.46 (2.02, 3.01) |
| Semaglutide vs SoC, GLP-1RAs | 1.66 (1.37, 2.03) | 1.38 (1.12, 1.70) |
| AS, As-started; AT, As-treated, Semaglutide, Injectable Semaglutide; SoC, Standard of care medications  DPP-4is, Dipeptidyl peptidase-4 inhibitors; SGLT-2is, Sodium-glucose Cotransporter-2 Inhibitors;  SUs, Sulfonylurea (SUs); GLP-1Ras, Glucagon-like peptide-1 agonists excluding semaglutide injectable  *Imputation method explained in Supplement method 1 and adjusted for baseline A1C as in the trial | | |

| Supplement Table 8: Results from sensitivity analysis (excluding patients with MCT), primary outcome (A1C <7%) | | |
| --- | --- | --- |
|  | AS analysis  *(1,135 matched pairs)* | AT analysis  *(1,137 matched pairs)* |
| Excluded due to MCT in the baseline | 102 | 82 |
| Risk ratio*† | 1.48 (1.32, 1.66) | 1.49 (1.37, 1.64) |
| MCT, Medullary carcinoma thyroid; AS, As-started; AT, As-treated, Semaglutide, Injectable Semaglutide; SoC, Standard of care medications; After excluding patients with MCT in the baseline, we had 37,580 unmatched patients in AS analysis and 29,616 unmatched patients in AT analysis  * The analysis results are from the propensity-score-matched imputed dataset, steps explained in supplement methods1  ^†^ Calculated from the marginal 2x2 table after imputing missing outcomes | | |

Supplement Table 9: Results from delta-adjusted Missing Not at Random (MNAR) analysis, in As-Started cohort for primary outcome (A1C <7%)

| Delta adjustment | Risk ratio (95% CI) * |
| --- | --- |
| -2.5 % | 1.15 (1.08, 1.23) |
| -2.0 % | 1.18 (1.10, 1.27) |
| -1.5 % | 1.21 (1.11, 1.32) |
| -1.0 % | 1.25 (1.14, 1.38) |
| -0.5 % | 1.30 (1.17, 1.44) |
| 0.0 % | 1.34 (1.19, 1.50) |
| 0.5 % | 1.36 (1.21, 1.52) |
| 1.0 % | 1.36 (1.20, 1.55) |
| 1.5 % | 1.35 (1.18, 1.55) |
| 2.0 % | 1.34 (1.17, 1.54) |
| 2.5 % | 1.33 (1.16, 1.52) |
| * The results are from the propensity-score-matched imputed dataset, steps explained in supplement methods 2 | |

Supplement Method 1: Multiple imputation using MissForest for the Primary analysis

1. We conducted a few diagnostics before imputing the A1C values.
   1. Because the outcome assessment window starts 275 days after CED, we compared the baseline characteristics between the patients who initiated semaglutide or standard of care but were censored before the start of the outcome assessment window to those who remained in the analytic cohort.
   2. We also checked the standardized difference, p-value, and area under the curve (AUC) in baseline characteristics value between patients who had and did not have A1C values in the outcome assessment window using the structural missing data investigations (SMDI) R package. This is one recommended diagnostic within a structured approach to assess the plausibility of missing at random (MAR) assumptions in real-world data settings. As noted in the original SMDI framework, the presence of measurable imbalance in patient characteristics between A1C observed and unobserved groups supports the plausibility of MAR assumptions. Based on the results from the above analysis, we observed some of the variables have standardized difference >0.1, Hoteling/Little p-value <0.001, and AUC > 0.5, which together suggests a missing at random (MAR)(8) mechanism.
   3. Link to R code code: <https://osf.io/j7h2q> and <https://osf.io/ut4w3>
   4. Link to file with results from the above diagnostics: <https://osf.io/pzm9j>
2. After diagnostics suggested that the missing A1C values were ~MAR, we implemented the following steps.
   1. We selected predictors for multiple imputation, where the predictors included all measured baseline covariates. We added a restriction to always include variables with a standardized difference greater than 0.1. We employed a non-parametric imputation approach using random forest algorithm to account for potential non-liner and non-additive relationships between covariates based on various simulation studies.(9, 10)
   2. A1C values were imputed based on the selected predictors using random forest methods in the unmatched dataset, resulting in 37 imputed datasets. We chose to create 37 imputed datasets because 37% of patients had missing A1C values(11).
   3. We created 1:1 nearest-neighbor propensity score (PS) matched cohorts using a 0.01 caliper across the 37 imputed datasets.
   4. We calculated c-statistics before and after PS matching.
   5. We calculated odds ratios, risk ratios, and changes in A1C values from baseline to follow-up where the estimates from imputed datasets were combined using Rubin’s rules.(12)
   6. Link to R code: <https://osf.io/45d2f> and list of predictors: https://osf.io/wdnf4
3. We applied the same procedures to perform multiple imputations for the as-treated cohort (secondary analysis) and the subgroup analyses. The key difference was adjusting the number of imputations based on the percentage of missing A1C values during the follow-up period.

Supplement Method 2: Sensitivity analyses, Alternative missing data imputation approaches

1. Tipping point analyses
   1. We calculated summary statistics for A1C values during the follow-up period, including the mean, median, 1st percentile, 10th percentile, 25th percentile, 75th percentile, 90th percentile, and 99th percentile for the entire matched cohort and separately for the exposure and comparator groups. The value at each of these percentiles was then imputed, and measures of effect were reported for both the intention-to-treat and as-treated cohorts.
2. Multiple imputation using chained equations (MICE):
   1. We assessed differences in baseline characteristics between those with and without A1C values during the follow-up period:
      1. Across all patients in the matched cohort
      2. Within the exposure group in the matched cohort
      3. Within the comparator group in the matched cohort
   2. We used a combined stepwise regression method to select predictors across three models:
      1. Stepwise logistic regression (p-entry = 0.10, p-removal = 0.15) with the presence or absence of follow-up A1C as the outcome and all covariates as predictors
      2. Stepwise linear regression (p-entry = 0.10, p-removal = 0.15) with A1C value during the follow-up period as the outcome and all covariates as predictors
      3. Stepwise linear regression (p-entry = 0.10, p-removal = 0.15) with baseline A1C value as the outcome and all covariates as predictors
   3. The selected variables from steps a and b were then used for multiple imputations, with the number of datasets imputed corresponding to the percentage of missing A1C values.
   4. We created 1:1 nearest-neighbor propensity score (PS) matched cohorts using a 0.01 caliper across the 37 imputed datasets.
   5. We calculated c-statistics before and after PS matching.
   6. We calculated odds ratios, risk ratios, and changes in A1C values from baseline to follow-up, where the estimates from imputed datasets were combined using Rubin’s rules.(12)
   7. Link to STATA code: <https://osf.io/zy69c>
3. Delta-adjusted missing not at random (MNAR) analysis:

To assess the robustness of the primary analysis of the missing at random assumption, we conducted a delta-adjusted sensitivity analysis. Delta-adjustment in the context of multiple imputation by chained equations (MICE) refers to a procedure that modifies imputed data to assess how violations from the Missing at Random (MAR) assumption, specifically Missing not at Random (MNAR) mechanisms, might impact the results of a statistical analysis. This adjustment involves adding a user-specified value (delta), which shifts the imputed values, allowing the exploration of different plausible scenarios of how the missing data might behave differently from the observed data.

- 1. The covariates used in this sensitivity analysis, both for the the imputation model and the propensity score matching model were identical to those in the primary analysis.
  2. The missing A1C values in the follow-up are imputed using MICE and our outcome of interest is A1C < 7%.
  3. To simulate a Missing Not at Random (MNAR) mechanism:
     1. The linear predictor in the imputation model was adjusted by fixed delta values ranging from –2.5% to +2.5% in 0.5% increments.
     2. A positive delta assumed that patients with missing A1C tend to have higher values.
     3. A negative delta assumed that patients with missing values tend to have lower values.
  4. After delta adjustment, a new binary outcome variable was created to reflect whether each patient’s adjusted A1C < 7%.
  5. The adjusted binary outcome variable was used to re-estimate the risk ratio, assessing the robustness of the results under MNAR assumptions.
  6. Link to R code: https://osf.io/njkgc

References

1. Schneeweiss S, Patorno E. Conducting Real-world Evidence Studies on the Clinical Outcomes of Diabetes Treatments. Endocr Rev. 2021;42(5):658-90.

2. Buse JB, Nordahl Christensen H, Harty BJ, Mitchell J, Soule BP, Zacherle E, et al. Study design and baseline profile for adults with type 2 diabetes in the once-weekly subcutaneous SEmaglutide randomized PRAgmatic (SEPRA) trial. BMJ Open Diabetes Res Care. 2023;11(3).

3. Ozempic (semaglutide) injection, for subcutaneous use. Prescribing Information. Silver Spring, MD2017.

4. GLUCOPHAGE and GLUCOPHAGE XR. Prescribing information. Silver Spring, MD2018.

5. Sun JW, Rogers JR, Her Q, Welch EC, Panozzo CA, Toh S, et al. Adaptation and Validation of the Combined Comorbidity Score for ICD-10-CM. Med Care. 2017;55(12):1046-51.

6. Gagne JJ, Glynn RJ, Avorn J, Levin R, Schneeweiss S. A combined comorbidity score predicted mortality in elderly patients better than existing scores. J Clin Epidemiol. 2011;64(7):749-59.

7. Kim D, Schneeweiss S, Glynn R, Lipsitz L, Rockwood K, Avorn J. Measuring Frailty in Medicare Data: Development and Validation of a Claims-Based Frailty Index. Journals of Gerontology: Medical Sciences. 2017;73:980-7.

8. Weberpals J, Raman SR, Shaw PA, Lee H, Hammill BG, Toh S, et al. smdi: an R package to perform structural missing data investigations on partially observed confounders in real-world evidence studies. JAMIA Open. 2024;7(1):ooae008.

9. Karter AJ, Warton EM, Moffet HH, Ralston JD, Huang ES, Miller DR, et al. Revalidation of the Hypoglycemia Risk Stratification Tool Using ICD-10 Codes. Diabetes Care. 2019;42(4):e58-e9.

10. Shah AD, Bartlett JW, Carpenter J, Nicholas O, Hemingway H. Comparison of random forest and parametric imputation models for imputing missing data using MICE: a CALIBER study. Am J Epidemiol. 2014;179(6):764-74.

11. Buuren Sv. How many imputations? Flexible Imputation of Missing Data. 2nd ed. ed2018.

12. Rubin DB. Multiple Imputation for Nonresponse in Surveys: John Wiley & Sons, Inc.; 1987.
